# The Efficacy of Mesenchymal Stem Cell Therapies in Rodent Models of Multiple Sclerosis: An Updated Systematic Review and Meta-Analysis

**DOI:** 10.3389/fimmu.2021.711362

**Published:** 2021-08-26

**Authors:** Jialin He, Yan Huang, Jianyang Liu, Ziwei Lan, Xiangqi Tang, Zhiping Hu

**Affiliations:** ^1^Department of Neurology, The Second Xiangya Hospital, Central South University, Changsha, China; ^2^National Health Commission Key Laboratory of Birth Defect for Research and Prevention, Hunan Provincial Maternal and Child Health Care Hospital, Changsha, China

**Keywords:** multiple sclerosis, mesenchymal stem cell, remyelination, meta-analysis, animal model

## Abstract

Studies have demonstrated the potential of mesenchymal stem cell (MSC) administration to promote functional recovery in preclinical studies of multiple sclerosis (MS), yet the effects of MSCs on remyelination are poorly understood. We wished to evaluate the therapeutic effects of MSCs on functional and histopathological outcomes in MS; therefore, we undertook an updated systematic review and meta-analysis of preclinical data on MSC therapy for MS. We searched mainstream databases from inception to July 15, 2021. Interventional studies of therapy using naïve MSCs in *in vivo* rodent models of MS were included. From each study, the clinical score was extracted as the functional outcome, and remyelination was measured as the histopathological outcome. Eighty-eight studies published from 2005 to 2021 met the inclusion criteria. Our results revealed an overall positive effect of MSCs on the functional outcome with a standardized mean difference (SMD) of −1.99 (95% confidence interval (CI): −2.32, −1.65; p = 0.000). MSCs promoted remyelination by an SMD of −2.31 (95% CI: −2.84, −1.79; p = 0.000). Significant heterogeneity among studies was observed. Altogether, our meta-analysis indicated that MSC administration improved functional recovery and promoted remyelination prominently in rodent models of MS.

## Introduction

Multiple sclerosis (MS) is a devastating disease that presents in young adults and can cause progressive physical disability and cognitive impairment (1). The etiology of MS is not known, but its pathophysiology has been reported to be associated with the formation of autoreactive lymphocytes and antigen-presenting cells in the body. The inflammatory cytokines generated by these cells in turn promote the recruitment of immune cells in the central nervous system (CNS), which destroy myelin sheaths and axon neurons, contributing to inflammation, demyelination, and massive loss of neurons in the CNS (2).

MS comprises mainly a relapsing–remitting phase and a progressive phase (3). Better understanding regarding the mechanisms of relapsing–remitting MS (RRMS) has led to the development of several disease-modifying therapeutic regimens that reduced the frequency and severity of acute relapses through direct action on the immune system, while progression in the treatment of progressive MS (PMS) remains relatively lacking (4). In general, the exploitation regarding new treatment options that promote favorable CNS repair in MS will be of great significance and is promising.

Potent immunomodulatory properties and pleiotropic effects have made mesenchymal stem cell (MSC) candidates for MS treatment (5–8). Abundant data have indicated that transplantation of MSCs may be efficacious in animal models of MS. Studies have shown that MSCs can induce autoantigen immunotolerance, enhance remyelination, maintain and remodel axons, and promote functional improvement in animal models of MS (9). Conversely, other studies have reported that MSC administration did not ameliorate the neurological deficit of MS in animal models (10, 11). These differences may be related to the cell type, transplantation route, and dose of MSCs.

As the questions about the optimal pattern of MSC therapy in MS remain unanswered, the analysis that evaluates the overall therapeutic effect of MSCs in animal models of MS among different experimental conditions will be prospective. And such analysis is also necessary for the guidance of clinical translation. A recent meta-analysis by Yang and colleagues demonstrated the functional promotion effects of MSC transplantation in the experimental autoimmune encephalomyelitis (EAE) model of MS, but the database was only until October 1, 2017 (12). In addition, the meta-analysis that investigates the histopathological efficiency of MSC transplantation for MS is also lacking. Myelin repair-promoting therapy has been recognized as a well-acknowledged approach to prevent disease progression of MS, and myelin regeneration is increasingly measured as the structure outcomes of several studies (13–15). Thus, remyelination was measured as the histopathological outcome in our meta-analysis.

The aim of our study was to provide evidence relating to the therapeutic effects of MSCs on the functional and histopathological outcomes in rodent models of MS, through performing an updated systematic review and meta-analysis.

## Materials and Methods

The present meta-analysis followed the guidelines of the Preferred Reporting Items for Systematic Reviews and Meta-Analyses (PRISMA) checklist (**Supplementary Table 1**) (16).

### Search Strategy

We searched PubMed, Embase, and Web of Science databases from inception to July 15, 2021, using the following search strategy: (“mesenchymal stem cells” OR “mesenchymal stromal cells” OR “mesenchymal stem cell” OR “mesenchymal stromal cell”) AND (“Multiple sclerosis” OR “MS” OR “Experimental Autoimmune Encephalomyelitis” OR “Experimental Allergic Encephalomyelitis”). Besides, the reference lists of eligible studies were also reviewed to identify other relevant articles.

### Inclusion and Exclusion Criteria

The eligibility criteria were set up according to the PICOS scheme (population, intervention, control, outcome and study design) (17). Studies were included if they met the following criteria: i) a rodent model of MS was induced; ii) the effect of unmodified MSCs was tested in at least one experimental group; iii) studies provided adequate data on clinical scores or remyelination; iv) experimental studies were presented in original research and published in peer-reviewed journals; and v) studies are published in English.

The exclusion criteria were as follows: i) studies that did not involve *in vivo* testing; ii) the outcome did not include the clinical score or remyelination; iii) cells were administered before the animal model induction; iv) studies that used only extracellular vesicles (EVs), conditioned medium derived from MSCs, or differentiated MSCs; and v) studies that reported sample size incompletely.

### Study Selection

After removal of duplicated data, two independent investigators screened titles and abstracts for inclusion. Non-relevant studies were excluded if the two researchers agreed. Those deemed “potentially relevant studies” were recorded, and the full text was acquired. Then, the two researchers screened the full-text articles independently to evaluate the final eligibility according to the inclusion and exclusion criteria stated above. Disagreement was addressed by discussion with a third investigator, if needed.

### Data Collection

The following items from the eligible studies were extracted and documented independently by two investigators: general information (first author, publication year, and country); experimental methods (numbers of animals per group for individual comparisons; species and strain of animals; gender; methods of MS induction in the animal model; sources and types of MSCs; dose of MSCs; delivery route of MSCs; time of administration [days post immunization (DPI)]; duration of follow-up; functional outcome (clinical score); and histopathological outcome (remyelination).

Data regarding the mean and standard deviation (SD) of a particular parameter from the MSC-treatment group and control group were extracted independently by the two researchers. If data were presented only graphically, the mean and SD from graphs were measured using a “digital ruler” (Graph Digitizer 2.26; GetData; http://getdata-graph-digitizer.com/download.php/). If SD was not reported, we calculated it through multiplying the standard error (SE) by the square root of the group size. If a control group served more than one experimental group differentiated by a different dose, delivery route, or administration time, then the size of control group was divided by the number of experimental groups served. Moreover, if an outcome observation was carried out at different times, only the data from the longest time were extracted. Disagreement between two investigators was solved by checking the data in the publications together.

### Methodological Quality of Studies

The quality of each study was assessed according to the Collaborative Approach to Meta-Analysis and Review of Animal Data from Experimental Studies (CAMARADES) checklist. This comprises i) publication in a peer-reviewed journal; ii) reporting of a sample size calculation; iii) allocation of randomized treatment; iv) allocation concealment; v) blind assessment of outcome; vi) use of suitable animal models; vii) avoidance of anesthetic agents with significant intrinsic neuroprotective activity (e.g., ketamine); viii) statement of compliance with regulatory requirements; ix) statements describing temperature control; and x) declarations of potential conflicts of interest. Therefore, the total score of each study ranged from 0 through 10, where “10” represents the greatest methodological strength. The sum of the quality scores was recorded for each research by the two investigators independently.

### Statistical Analysis

The meta-analysis was carried out using Stata 15.1 (StataCorp, College Station, TX, USA) and Cochrane Review Manager 5.3 (Cochrane Collaboration; www.cochrane.org/). The combined effect size was calculated as standardized mean differences (SMDs) with 95% confidence interval (CI) between MSC-treatment group and control group. Hedge’s statistic was used to estimate the effect size (18). Forest plots were generated to display the SMD and 95% CI of each study, and the pooled mean difference by combining all studies. p-Value < 0.05 was considered statistically significant.

Statistical heterogeneity was assessed using the *I* ^2^ statistic. *I* ^2^ that ranged from 0% to 40%, 30% to 60%, 50% to 90%, and 75% to 100% was defined as “low,” “moderate,” “substantial,” and “considerable” heterogeneity, respectively (19). A random-effects model was used if substantial heterogeneity (*I* ^2^ > 50%, p < 0.05) was observed. Sensitivity analyses were undertaken to remove extreme values thought to be driving the overall effect. Subgroup analysis and multivariate meta-regression analysis were carried out to identify the source of the heterogeneity.

Publication bias was detected using funnel plots (20). Asymmetry was evaluated through Egger’s test and “trim-and-fill” method (21).

## Results

### Study Selection

**Figure 1:**
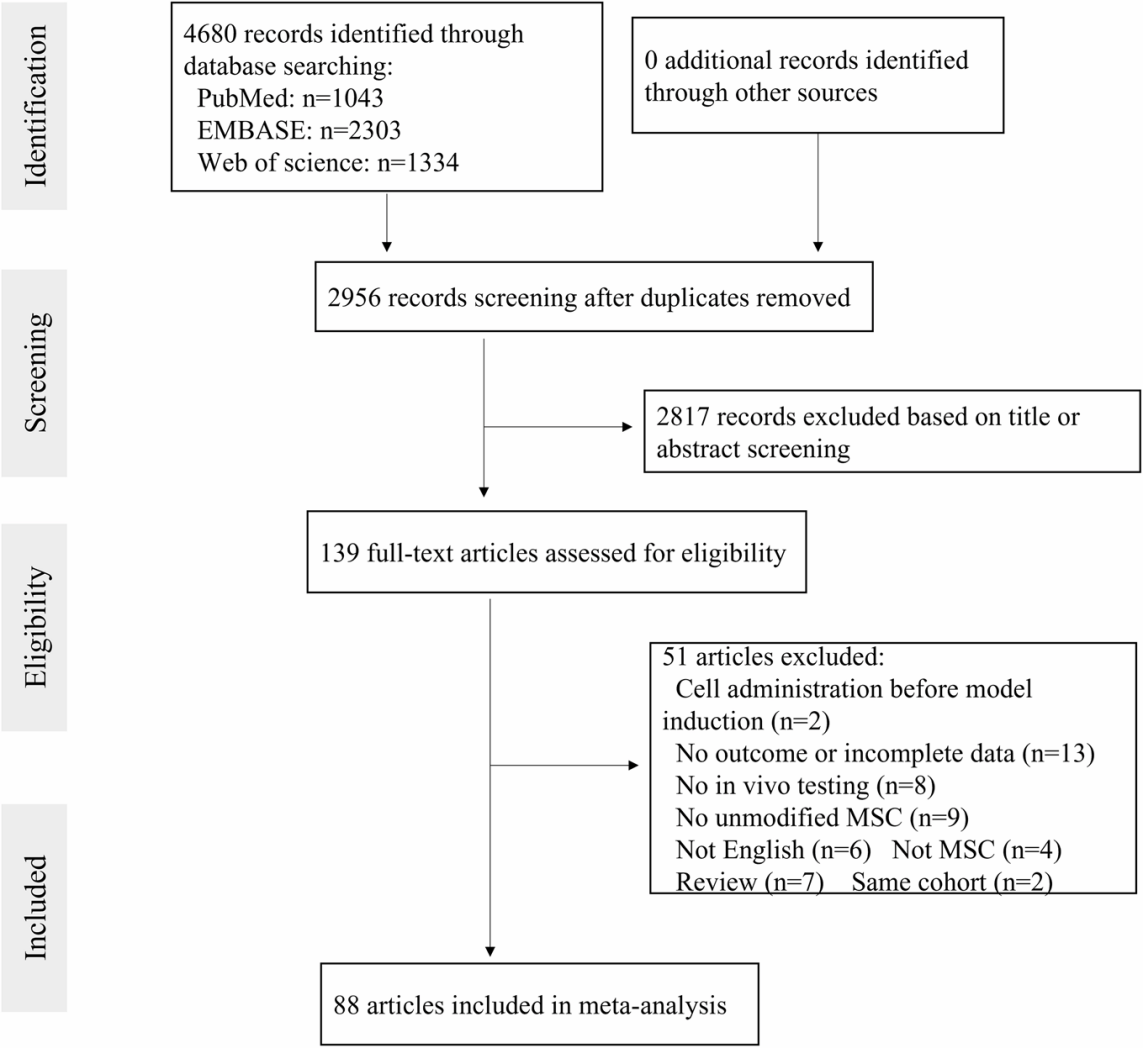
The flow diagram describing literature search and study selection.

The literature search identified 4,680 potential studies at the primary retrieval: 1,043 records in PubMed, 2,303 in Embase, and 1,334 in Web of science. After review and exclusion, 139 full-text articles remained and were evaluated for inclusion eligibility. From these, 51 records were excluded due to the reasons given in **Figure 1**. Finally, data from 88 studies published from 2005 to 2021 were included in the meta-analysis.

### Study Characteristics

The overall study characteristics are outlined in **Supplementary Table 2**, and the reference lists of included studies are summarized in the **Supplementary Materials**. All research was undertaken in rats and mice, and female animals were used in most of the studies. The animal model of MS was induced *via* myelin oligodendrocyte glycoprotein (MOG), proteolipid protein (PLP), guinea pig spinal cord homogenate (GPSCH), myelin basic protein (MBP), or cuprizone, with MOG accounting for the greatest proportion. The vast majority of studies used bone marrow-derived MSC (BM-MSC) and adipose tissue-derived MSC (ASC), or umbilical cord MSC (UC-MSC) derived from mice, rats, or humans. The remaining studies used placenta-derived MSC (PMSC), embryonic stem cell-derived MSC (ES-MSC), amnion MSC (AMC), decidua-derived MSC (DMSC), periodontal ligament-derived MSC (PDLSC), and murine endometrial-derived MSC (meMSC). The number of MSCs also varied greatly among these studies, with 1 × 10^6^ being the most common, and those MSCs were transplanted *via* intravenous (IV) or intraperitoneal (IP) injection in most studies. Furthermore, MSCs were administered from 0 days to 13 weeks after MS induction, and the duration of follow-up ranged from 18 to >315 days.

Functional outcomes were assessed in 75 studies using clinical score. In addition, the Morris water maze, basket test, footprint analysis, track visualizations, and Rotarod™ experiments were undertaken to measure functional outcomes in some publications. Forty-eight studies evaluated the histopathological outcomes (remyelination) using transmission electron microscope (TEM), Luxol Fast Blue (LFB) staining, MBP staining, Spielmeyer staining, Solochrome Cyanine staining, hematoxylin and eosin (H&E) staining, or magnetic resonance imaging (MRI). Intriguingly, six studies measured the oligodendrocyte precursor cell (OPC) count, and nine reports detected the oligodendrocyte count (**Supplementary Table 3**).

### Study Quality

The quality assessment of the included studies is outlined in **Table 1**; the detailed information of each study is listed in **Supplementary Table 4**. The quality score of the studies ranged from 2 to 8. All studies were published in peer-reviewed journals and used suitable animal models. Also, 85.23% of studies reported a statement of compliance with regulatory requirement. Only 2.27% reported a calculation for the sample size, 39.77% of studies reported allocation of randomized treatment, 11.36% reported allocation concealment, and 42.05% of studies declared a blind assessment of outcome. In addition, the percentage of studies that avoided neuroprotective anesthetic agents, stated a temperature control, and a declared conflict of interest was 6.82%, 15.91%, and 70.45%, respectively.

**Table 1:** Percentage of included studies satisfying each criterion of CAMARADES checklist.

Quality score criterion	Percentage of qualified studies
Publication in a peer-reviewed journal	100%
Reporting of a sample size calculation	2.27%
Randomized treatment allocation	39.77%
Allocation concealment	11.36%
Blind assessment of outcome	42.05%
Use of suitable animal models	100%
Avoidance of anesthetics with neuroprotective activity	6.82%
Statement of regulatory requirements	85.23%
Statements describing temperature control	15.91%
Declarations of potential conflicts of interest	70.45%

CAMARADES, Collaborative Approach to Meta-Analysis and Review of Animal Data from Experimental Studies.

### Meta-Analysis

**Figure 2:**
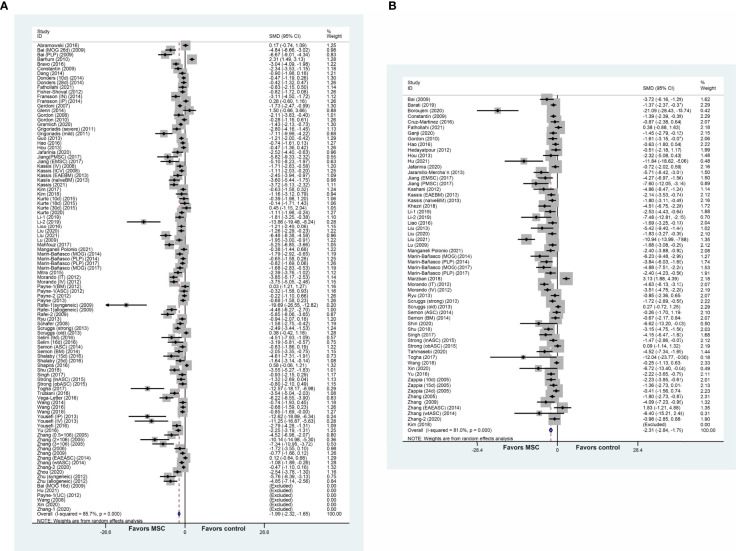
Forest plot shows the mean effect size and 95% confidence interval (CI) for **(A)** clinical score and **(B)** remyelination between MSC treatment group and control group in all studies. The mean value or standard deviation (SD) in Bai et al. (22), Hu et al. (23), Payne et al. (10), Wang et al. (24), Xin et al. (25), Zhang et al. (26), and Kim et al. (27) was 0. MSC, mesenchymal stem cell; SMD, standardized mean difference; MOG, myelin oligodendrocyte glycoprotein.

MSC administration exhibited a favorable effect on the clinical score and remyelination. More specifically, the composite weighted mean of clinical score was −1.99 (95% CI: −2.32 to −1.65, p = 0.000, 75 studies, 101 comparisons; **Figure 2A**), and remyelination was −2.31 (95% CI: −2.84 to −1.79, p = 0.000, 48 studies, 59 comparisons; **Figure 2B**). We also undertook a pooled analysis for the OPC count and oligodendrocyte count: the composite weighted mean was −3.66 (95% CI: −6.44 to −0.88, p = 0.000, six studies, six comparisons) (**Supplementary Figure 1A**) and −3.64 (95% CI: −5.14 to −2.14, p = 0.000, nine studies, nine comparisons) (**Supplementary Figure 1B**), respectively. These findings provided evidence of the advantageous effect of MSCs on MS models. The *I* ^2^ statistic suggested remarkable heterogeneity among comparisons of clinical score and remyelination (p = 0.000).

### Sensitivity Analysis

**Figure 3:**
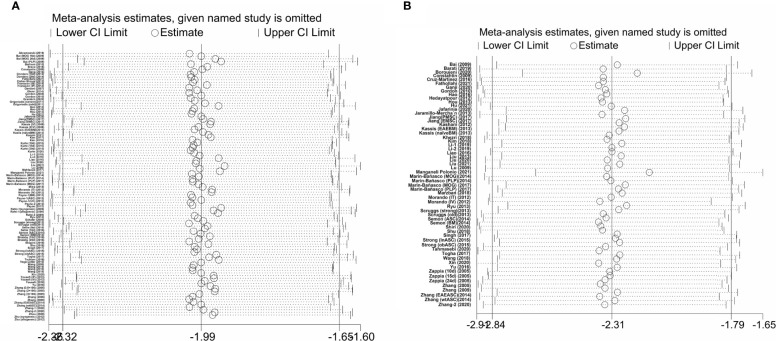
Sensitivity analysis of the studies included in clinical score **(A)** and remyelination **(B)**.

We performed a sensitivity analysis to identify the stability of results by sequential omission of each study because of the notable heterogeneity. As depicted in **Figures 3A, B**, the pooled SMD of clinical score and remyelination was not affected by any study.

### Stratified Analysis and Meta-Regression Analysis

We did a further subgroup analysis for the clinical score and remyelination based on different categories, which are summarized in **Tables 2** and **3**, respectively. In general, the prominent efficacy of MSCs intervention was observed in most subgroups, but significance was not seen in a few individual subgroups (p ≥ 0.05). Significant differences between groups were found in some stratified analyses, but the source of heterogeneity was not identified.

**Table 2:** Stratified meta-analysis of clinical score.

Categories	No. of studies	Pooled SMD (95% CI)	p-Value	Heterogeneity test			Between groups p-value
				Q statistics	*I* ^2^	p-Value	
**Species**							0.28
Mice	84	−1.93 (−2.29, −1.58)	0.000	605.43	86.3%	0.000	
Rats	11	−2.57 (−3.67, −1.46)	0.000	52.74	81.0%	0.000	
**Gender**							0.01
Female	81	−1.73 (−2.06, −1.40)	0.000	501.10	84.0%	0.000	
Male	6	−4.37 (−6.36, −2.38)	0.000	33.82	85.2%	0.000	
NR	8	−3.00 (−4.59, −1.41)	0.000	68.02	89.7%	0.000	
**Methods of MS induction**							0.03
MOG	81	−1.79 (−2.14, −1.45)	0.000	541.85	85.2%	0.000	
PLP	9	−2.81 (−4.02, −1.59)	0.000	55.52	85.6%	0.000	
GPSCH	5	−5.51 (−8.90, −2.12)	0.001	48.81	91.8%	0.000	
MBP	NA	NA	NA	NA	NA	NA	
**MSC source**							0.005
Xenogeneic	50	−1.66 (−2.09, −1.23)	0.000	334.25	85.3%	0.000	
Syngeneic	37	−2.05 (−2.60, −1.49)	0.000	236.99	84.8%	0.000	
Allogeneic	8	−4.09 (−5.50, −2.68)	0.000	39.54	82.3%	0.000	
**MSC type**							0.0002
BM-MSC	56	−2.37 (−2.87, −1.87)	0.000	463.43	88.1%	0.000	
DMSC	1	−3.04 (−4.10, −1.98)	0.000	NA	NA	NA	
ASC	22	−1.33 (−1.84, −0.82)	0.000	84.72	75.2%	0.000	
UC-MSC	7	−1.22 (−1.91, −0.52)	0.001	21.62	72.2%	0.001	
PMSC	5	−2.07 (−3.89, −0.26)	0.025	28.59	86.0%	0.000	
ES-MSC	2	−2.67 (−6.92, 1.58)	0.217	6.53	84.7%	0.011	
PDLSC	1	−3.54 (−5.04, −2.03)	0.000	NA	NA	NA	
meMSC	1	−0.38 (−1.44, 0.69)	0.487	NA	NA	NA	
**Dose**							<0.00001
>1 × 10^6^	24	−2.37 (−3.16, −1.58)	0.000	211.26	89.1%	0.000	
≤1 × 10^6^	70	−1.82 (−2.18, −1.46)	0.000	412.39	83.2%	0.000	
NR	1	−6.48 (−8.38, −4.58)	0.000	NA	NA	NA	
**Delivery route**							<0.00001
IV	56	−1.83 (−2.20, −1.47)	0.000	265.02	79.2%	0.000	
IP	26	−2.15 (−2.88, −1.42)	0.000	215.82	88.4%	0.000	
IN	3	−2.06 (−3.24, −0.89)	0.001	5.70	64.9%	0.058	
ICV	8	−2.70 (−4.62, −0.77)	0.006	114.49	93.9%	0.000	
IM	1	0.58 (−0.06, 1.21)	0.074	NA	NA	NA	
IT	1	−3.85 (−5.17, −2.53)	0.000	NA	NA	NA	
**Administration time**							0.03
>14 days	20	−2.76 (−3.74, −1.78)	0.000	149.70	87.3%	0.000	
≤14 days	70	−1.73 (−2.08, −1.37)	0.000	468.37	85.2%	0.000	
NR	5	−4.23 (−6.93, −1.54)	0.002	30.24	86.8%	0.000	
**Duration of Follow-up**							0.08
30–60 days	71	−1.86 (−2.24, −1.48)	0.000	466.51	85.0%	0.000	
<30 days	15	−1.91 (−2.81, −1.02)	0.000	131.53	89.4%	0.000	
>60 days	9	−3.33 (−4.57, −2.09)	0.000	44.76	82.1%	0.000	

NR, not recorded; MOG, myelin oligodendrocyte glycoprotein; PLP, proteolipid protein; GPSCH, guinea pig spinal cord homogenate; MBP, myelin basic protein; BM-MSC, bone marrow-derived mesenchymal stem cell; DMSC, decidua-derived mesenchymal stem cell; ASC, adipose tissue-derived mesenchymal stem cell; UC-MSC, umbilical cord mesenchymal stem cell; PMSC, placenta-derived mesenchymal stem cell; ES-MSC, embryonic stem cell-derived mesenchymal stem cell; PDLSC, periodontal ligament-derived mesenchymal stem cell; meMSC, murine endometrial-derived mesenchymal stem cell; IV, intravenous; IP, intraperitoneal; IN, intranasal; ICV, intra-cerebroventricular; IM, intramuscular; IT, intrathecal; NA, not available.

**Table 3:** Stratified meta-analysis of remyelination.

Categories	No. of studies	Pooled SMD (95% CI)	p-Value	Heterogeneity test			Between groups p-value
				Q statistics	*I* ^2^	p-Value	
**Species**							0.21
Mice	52	−2.21 (−2.76, −1.66)	0.000	275.35	81.4%	0.000	
Rats	6	−3.51 (−5.48, −1.54)	0.000	23.59	78.8%	0.000	
**Gender**							0.02
Female	39	−1.70 (−2.15, −1.24)	0.000	111.18	65.5%	0.000	
Male	13	−4.41 (−6.52, −2.31)	0.000	150.59	92.0%	0.000	
NR	6	−2.84 (−4.22, −1.50)	0.000	21.88	77.2%	0.000	
**Methods of MS induction**							0.33
MOG	39	−2.05 (−2.60, −1.49)	0.000	166.04	77.0%	0.000	
Cuprizone	10	−2.97 (−4.98, −0.96)	0.004	96.20	90.6%	0.000	
GPSCH	5	−4.36 (−7.17, −1.55)	0.002	22.66	82.3%	0.000	
PLP	4	−2.53 (−3.57, −1.48)	0.000	4.26	29.6%	0.235	
**MSC source**							<0.0001
Xenogeneic	24	−1.49 (−2.18, −0.80)	0.000	122.22	81.2%	0.000	
Syngeneic	30	−2.80 (−3.58, −2.01)	0.000	141.95	79.6%	0.000	
Allogeneic	4	−5.24 (−2.84, −1.79)	0.000	1.77	0.0%	0.622	
**MSC type**							0.01
BM-MSC	32	−2.74 (−3.53, −1.95)	0.000	201.50	84.6%	0.000	
ASC	17	−1.38 (−2.11, −0.64)	0.000	54.56	70.7%	0.000	
ES-MSC	1	−4.27 (−6.97, −1.56)	0.002	NA	NA	NA	
PMSC	1	−7.60 (−12.05, −3.15)	0.001	NA	NA	NA	
UC-MSC	5	−2.02 (−3.76, −0.27)	0.024	12.82	68.8%	0.012	
AMC	1	−3.15 (−4.75, −1.56)	0.000	NA	NA	NA	
meMSC	1	−2.40 (−3.89.−0.92)	0.000	NA	NA	NA	
**Dose**							<0.00001
>1 × 10^6^	13	−2.79 (−4.44, −1.15)	0.001	89.98	86.7%	0.000	
≤1 × 10^6^	44	−2.04 (−2.53, −1.56)	0.000	168.65	74.4%	0.000	
NR	1	−10.94 (−14.00, −7.88)	0.000	NA	NA	NA	
**Delivery route**							0.001
IV	33	−2.33 (−2.88, −1.77)	0.000	116.40	72.4%	0.000	
ICV	6	−3.23 (−5.03, −1.43)	0.000	17.35	71.2%	0.004	
IN	3	−5.19 (−10.08, −0.30)	0.037	36.12	94.5%	0.000	
ICP	1	−5.71 (−8.42, −3.01)	0.000	NA	NA	NA	
IP	14	−1.13 (−2.31, 0.05)	0.060	81.59	84.1%	0.000	
IT	1	−4.63 (−6.13, −3.13)	0.000	NA	NA	NA	
**Administration time**							0.46
>14 days	18	−1.88 (−2.91, −0.85)	0.000	115.48	85.3%	0.000	
≤14 days	37	−2.46 (−3.08, −1.85)	0.000	158.94	77.3%	0.000	
NR	3	−3.18 (−5.21, −1.15)	0.002	6.13	67.3%	0.047	
**Duration of Follow-up**							0.36
30–60 days	44	−2.15 (−2.75, −1.55)	0.000	229.87	81.2%	0.000	
>60 days	9	−3.23 (−4.66, −1.80)	0.000	44.88	82.2%	0.000	
<30 days	5	−2.78 (−5.03, −0.52)	0.016	23.38	82.9%	0.000	

NR, not recorded; MOG, myelin oligodendrocyte glycoprotein; GPSCH, guinea pig spinal cord homogenate; PLP, proteolipid protein; BM-MSC, bone marrow-derived mesenchymal stem cell; ASC, adipose tissue-derived mesenchymal stem cell; ES-MSC, embryonic stem cell-derived mesenchymal stem cell; PMSC, placenta-derived mesenchymal stem cell; UC-MSC, umbilical cord mesenchymal stem cell; AMC, amnion mesenchymal stem cell; meMSC, murine endometrial-derived mesenchymal stem cell; IV, intravenous; ICV, intra-cerebroventricular; IN, intranasal; ICP, intra-cerebroparenchymal; IP, intraperitoneal; IT, intrathecal; NA, not available.

Subsequently, multivariate meta-regression analysis was employed to explore potential contributions to the heterogeneity of the items mentioned in **Tables 2** and **3**. For clinical score, the source of heterogeneity was not discovered, but the source of MSCs may be the cause of heterogeneity in remyelination (p = 0.022).

### Publication Bias

**Figure 4:**
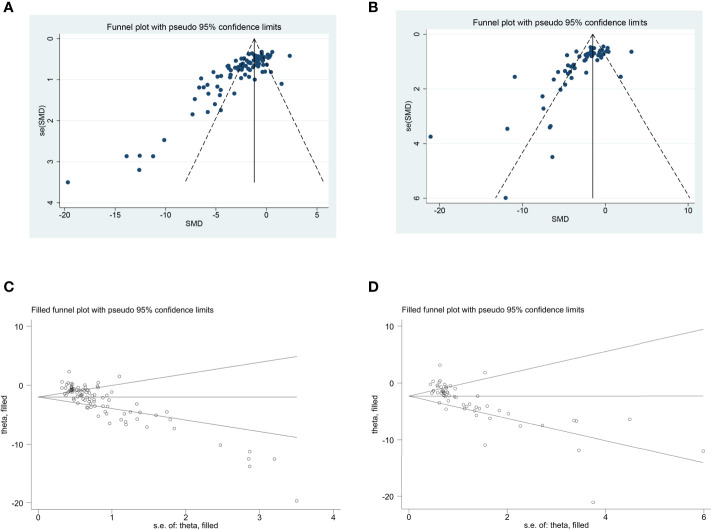
The evaluation of publication bias. Funnel plots for clinical score **(A)** and remyelination **(B)**. Each dot in the figure represents a study, with the y-axis signifying study quality and the x-axis showing the study results. **(C, D)** Trim-and-fill method was used to evaluate the missing studies in clinical score and remyelination. SMD, standardized mean difference.

The funnel plot was asymmetrical for the comparisons of the clinical score and remyelination (**Figures 4A, B**). Egger’s test demonstrated a conspicuous publication bias (p = 0.000). Then, we adopted the trim-and-fill approach to evaluate missing studies and recalculated the overall estimate of the pooled effect. Both of the imputed effect estimates of clinical score and remyelination were consistent with the previous one (SMD: −1.99, 95% CI: −2.32 to −1.65, p = 0.000; SMD: −2.31, 95% CI: −2.84 to −1.79, p = 0.000, respectively), which implied no “missing” studies (**Figures 4C, D**).

## Discussion

### Main Findings

Overall, our updated meta-analysis comprising 88 studies suggested that rodents with MS benefited from MSC therapy as manifested by significant amelioration of functional and histopathological outcomes. Subgroup analysis provided more detailed information.

#### Species, Gender, and Methods of MS Induction in Rodents

Commonly used experimental systems of MS includes EAE (28), virus-induced demyelination/inflammation (29), and toxin-induced demyelination models with cuprizone (30) or ethidium bromide (31). In general, distinct histopathological discrepancies are recognized among these models. EAE and virus-induced demyelination/inflammation model reproduce well the disseminated and inflammatory characteristics of MS, whereas toxin-induced demyelination models are more suitable for simulating the specific mechanism of myelin decline and regeneration (32).

EAE is induced in susceptible animal strains either through active immunization with CNS tissue or myelin peptides (e.g., MOG, GPSCH, PLP, and MBP) or by passive transfer of encephalitogenic T cells (33). C57BL/6 mice, SJL/J mice, NOD mice, Lewis rats, and Dark Agouti (DA) rats are frequently used rodent species. Individual models of EAE can resemble corresponding types of MS clinical forms. Immunization of SJL/J mice with PLP_139-151_ induces a relapsing–remitting disease course, while C57BL/6 mice often develop a chronic disease after being immunized with a high dose of MOG_35-55_. Interestingly, immunization of NOD mice with MOG_35-55_ results in an initial acute episode of EAE followed by a secondary progressive EAE course, which is reminiscent of the secondary progressive phase of MS patients (28, 34). And EAE displays a sex bias that parallels MS whereby females are more susceptible than males (35).

Animal studies can differ widely in terms of experimental conditions. Hence, the species, gender, and methods of MS induction in rodents were applied as the items of stratified analysis in our meta-analysis. No difference in the effect size of species was observed. Males seemed to be more sensitive to MSC treatment than females in terms of the clinical score and remyelination. MSC administration exhibited similar beneficial effects on remyelination among different MS models, but the rodents that were immunized with GPSCH responded best to MSC therapy in terms of the clinical score, followed by PLP immunization group and MOG immunization group. Those outcomes may be related to the small sample size.

#### Source and Type of MSCs

Several sources of MSCs have been used in experimental models of MS, but most studies used BM-MSCs or ASC from humans, mice, or rats (9). In general, previous data demonstrating the beneficial effects of MSCs were not dependent on the MSCs source (6). Zhu et al. observed no difference in the amelioration of EAE clinical signs between administration of syngeneic and allogeneic MSCs in animal models of MS (36). Similarly, another study suggested that PMSC and ES-MSC exhibited a similarly favorable effect on MS models (37). Payne et al. reported no beneficial effect of BM-MSC, ASC, and UC-MSC in animal models of MS when MSCs were administered after symptom onset (10). In our meta-analysis, the source of MSC was correlated with the effect size in clinical score and remyelination. Allogeneic MSCs displayed the greatest efficacy, followed by syngeneic MSCs and then xenogeneic MSCs. And the types of the MSC also had impact on the effect size in terms of the clinical score and remyelination; however, the number of studies included in several subgroups was too small, and larger preclinical studies are needed to explore this issue in-depth.

#### Dose and Delivery Route

Delivery dose and route are typically the topic of concern when MSC therapy is applied in clinical situations. The literature suggests that the MSC dose administered in rodent models of MS is in the range from 3 × 10^5^ to 1 × 10^7^. These doses exerted protective effects when administered alone or in multiple injections (6). Yousefi et al. attempted to compare the therapeutic impact of ASC injected *via* IP and IV routes but did not document the disparity in improvement of the clinical score using these two routes (38). Morando and colleagues also did not observe the significant difference when they compared the clinical course of mice IV injected with those treated intrathecally (IT) (39). The meta-analysis by Yang and colleagues did not find a significant difference in the effect size with regard to the dose and the delivery methods of MSCs (12). Inversely, we observed that a high dose of MSCs (>1 × 10^6^) improved the functional and histopathological outcomes better than a low dose (≤1 × 10^6^). Meanwhile, the delivery route contributed to apparent differences in the clinical score and remyelination. For the clinical score, IT injection seems to show the greatest efficacy, followed by intra-cerebroventricular (ICV), IP, intranasal (IN), and IV injections. In addition, ICV injection was discovered to be the most effective administration route to promote remyelination in comparison with IT, IV, or IP injection. IN and intra-cerebroparenchymal (ICP) injections were also favorable, but large CIs and small sample sizes diminished the robustness of the data.

#### Time of Administration and Duration of Follow-Up

It is also considerable to determine the role of MSCs in the light of the time of administration and duration of follow-up. In studies that explored the therapeutic effects of MSCs in MS models, MSCs were administered at the onset, peak, or after EAE stabilization. The optimal time of MSC transplantation was controversial. Several studies demonstrated the ability of MSCs to promote functional recovery regardless of the injection time (22, 40). Conversely, other research concluded that the beneficial effect of MSCs was negatively correlated with the time of administration. More specifically, MSC injection induced a significant improvement in the clinical score in animals suffering from EAE at the onset or peak of the disease, with more sustained effects being documented at disease onset (41, 42). Later administration of MSCs had little or no effect on clinical signs or remyelination (41, 43, 44). However, our meta-analysis showed a positive correlation between the effect size and administration time in the clinical score, but not for remyelination. And no difference in the effect size of follow-up duration was observed.

The findings of subgroup analysis are speculative because they are based on the reanalysis of published data, rather than the data from a well-designed randomized controlled trial. Therefore, although the subgroup analysis provided updated evidence, the results of subgroup analysis should be viewed with caution.

### Mechanisms of the MSC Therapies in MS

Studies have suggested that MSC transplantation may aid in neuroprotection, anti-inflammation, neuroregeneration, and tissue repair in animal models of MS (45, 46). MSCs confer benefit mainly *via* the release of soluble trophic factors that stimulate intrinsic tissue restoration mechanisms and the production of EV that enables cell-to-cell communication.

**Figure 5:**
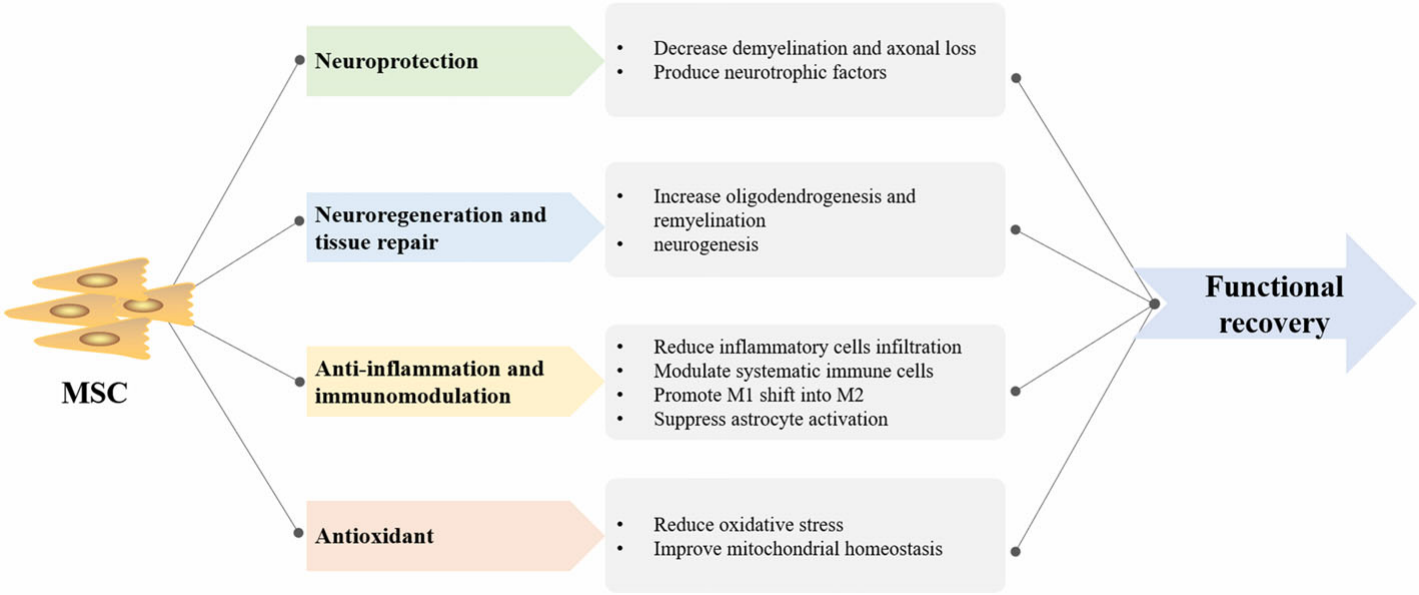
The summary of mesenchymal stem cell (MSC)-derived therapeutic mechanism in multiple sclerosis (MS). M1, proinflammatory subtypes of microglia; M2, anti-inflammatory subtypes of microglia.

MSC transplantation results in reduction of demyelination and axonal loss through production of neurotrophic factors, such as fibroblast glial factor (FGF), brain-derived neurotropic factor (BDNF), and neural growth factor (NGF) (47). Increased oligodendrogenesis, remyelination, and neurogenesis can be attributed to the neuroregeneration ability of MSCs. The anti-inflammation effect of MSCs not only aids the decline in the numbers of inflammatory cell infiltration but also modulates systematic immune cells (48). Barati et al. noted that MSC infusion diminished neuroinflammation by suppressing activation of astrocytes and microglia as well as promoting a shift of proinflammatory subtypes of microglia (M1) into anti-inflammatory microglia (M2) (49). In addition, another study demonstrated that MSCs attenuated MS by reducing oxidative stress and improving mitochondrial homeostasis (50). Overall, the mechanism of MSC-derived therapeutic effects in MS is depicted in **Figure 5**.

### Clinical Perspective

To date, a considerable number of small clinical trials involving MSCs in MS with inspiring results have been completed (51–56). Those trials have supported the safety, tolerability, and feasibility of MSC transplantation in MS disorders. Most studies used autologous BM-MSC, and allogeneic PMSC or UC-MSC was applied in a few trials. The infusion routes of MSCs were IV and IT. However, the optimal choice of the source, dose, and transplantation route of MSCs is inconclusive. Data have shown that systemic delivery of MSCs provided safer and beneficial effects (57), but a recent double-blind phase II trial involving 48 patients with PMS found that IT injection was more efficacious than IV injection for several parameters of the outcome (58). Intriguingly, another open prospective study, which was conducted by the same group, noted that multiple IT injections of autologous MSC in patients with PMS were shown safe at the short/intermediate term and induced clinical benefits (59). As many open issues are still unsolved, larger and longer-term trials are warranted to determine the more favorable parameter of MSC transplantation and to further evaluate the potential of cellular therapy in MS.

### Limitations

The conclusions from our systematic review were limited by several factors. Firstly, because of a lack of rigor in study design or methodology in parts of a particular study, our conclusions are tempered by the low quality of the studies included in our analysis. Secondly, all data were extracted from the eligible studies that published as original research; therefore, we did not gain access to unpublished results including conference abstract, and this may contribute to the obvious publication bias. Thirdly, there was considerable heterogeneity in statistical analysis across studies.

## Conclusion

In summary, our systematic review and meta-analysis provide a comprehensive, up-to-date evaluation of the available data concerning the efficacy of MSC therapy for MS in preclinical settings. MSC administration demonstrated a considerable beneficial effect with regard to functional outcome and histopathological outcome. Future preclinical studies should consider the study design and methodological rigor. A well-designed study will contribute to a better determination of the effect regarding MSC therapy in MS treatment.

## Data Availability Statement

The original contributions presented in the study are included in the article/**Supplementary Material**. Further inquiries can be directed to the corresponding author.

## Author Contributions

ZH supervised the project. JH, YH, and XT analyzed the data. JL and ZL extracted the data. JH and ZH wrote the paper. All authors contributed to the article and approved the submitted version.

## Funding

This work was supported by the National Natural Science Foundation of China [grant numbers 81974213, 81801188] and Natural Science Foundation of Hunan Province, China [grant number 2019JJ40421].

## Conflict of Interest

The authors declare that the research was conducted in the absence of any commercial or financial relationships that could be construed as a potential conflict of interest.

## Publisher’s Note

All claims expressed in this article are solely those of the authors and do not necessarily represent those of their affiliated organizations, or those of the publisher, the editors and the reviewers. Any product that may be evaluated in this article, or claim that may be made by its manufacturer, is not guaranteed or endorsed by the publisher.

## Abbreviations

MS, multiple sclerosis; CNS, central nervous system; RRMS, relapsing–remitting MS; PMS, progressive MS; MSCs, mesenchymal stem cells; BM-MSC, bone marrow-derived MSC; ASC, adipose tissue-derived MSC; UC-MSC, umbilical cord MSC; PMSC, placenta-derived MSC; EAE, experimental autoimmune encephalomyelitis; PRISMA, Preferred Reporting Items for Systematic Reviews and Meta-Analyses; EV, extracellular vesicles; DPI, days post immunization; SD, standard deviation; SE, standard error; CAMARADES, Collaborative Approach to Meta-Analysis and Review of Animal Data from Experimental Studies; SMD, standardized mean differences; CI, confidence interval; MOG, myelin oligodendrocyte glycoprotein; PLP, proteolipid protein; GPSCH, guinea pig spinal cord homogenate; ES-MSC, embryonic stem cell-derived MSC; AMC, amnion MSC; DMSC, decidua-derived MSC; PDLSC, periodontal ligament-derived MSC; meMSC, murine endometrial-derived mesenchymal stem cell; IV, intravenous; IP, intraperitoneal; ICV, intra-cerebroventricular; IN, intranasal; ICP, intra-cerebroparenchymal; IT, intrathecal; TEM, transmission electron microscope; LFB, Luxol Fast Blue; MBP, myelin basic protein; H&E, hematoxylin and eosin; MRI, magnetic resonance imaging; OPC, oligodendrocyte precursor cell; DA, Dark Agouti; FGF, fibroblast glial factor; BDNF, brain-derived neurotropic factor; NGF, neural growth factor; M1, promoting proinflammatory subtypes of microglia; M2, anti-inflammatory microglia.
