# Supplementary material for: The Efficacy of Mesenchymal Stem Cell Therapies in Rodent Models of Multiple Sclerosis: An Updated Systematic Review and Meta-Analysis
Source: Front Immunol. 2021 Aug 26;12:711362. doi: 10.3389/fimmu.2021.711362 (PMC8427822; doi:10.3389/fimmu.2021.711362)
Supplement: Supplementary file 1 [file DataSheet_1.pdf]

Supplementary Material

Supplementary Figures and Tables

Supplementary Figures

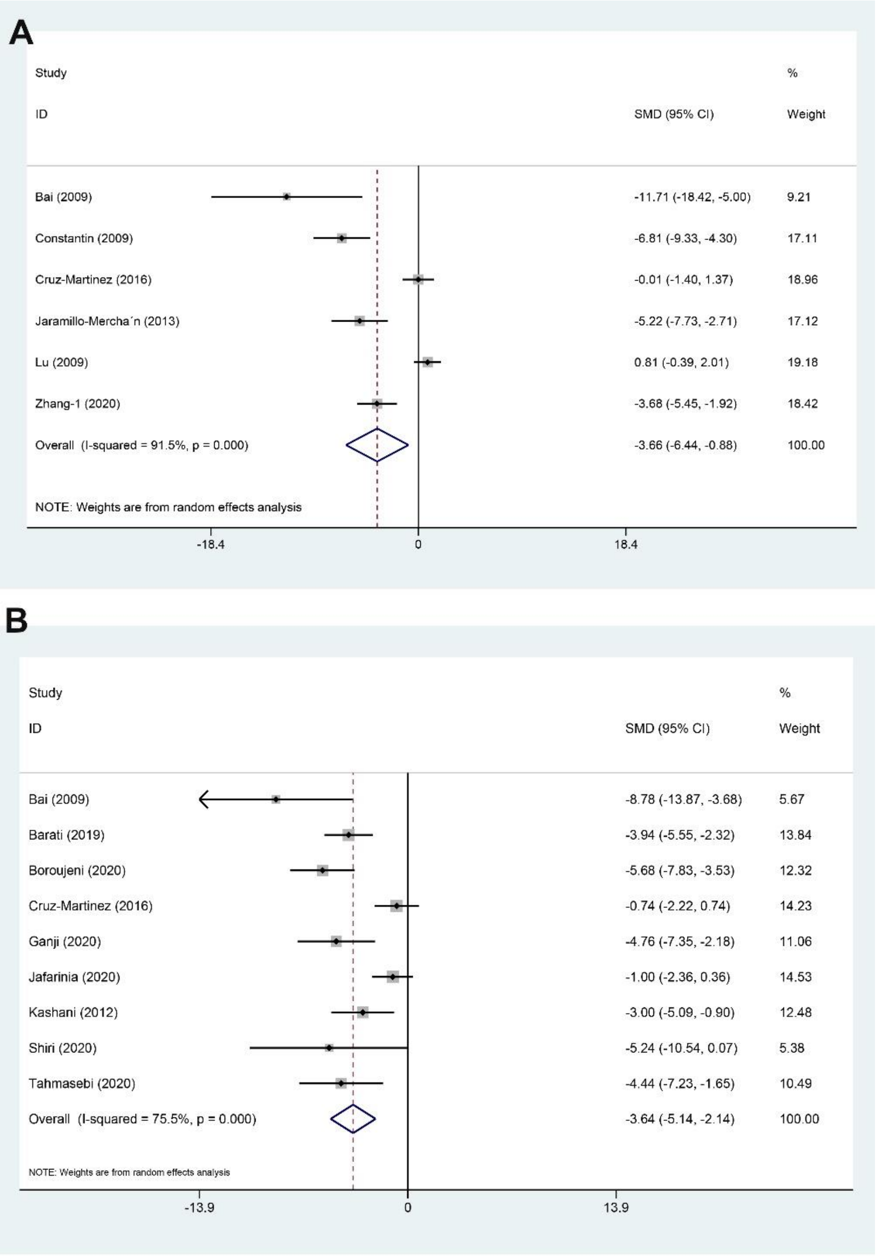

Supplementary Figure 1. Effect size of included comparisons. Forest plot showed mean effect size and 95 % confidence interval (CI) for (**A**) oligodendrocyte precursor cell (OPC) counts and (**B**) oligodendrocyte counts.

Supplementary Tables

Supplementary Table 1. PRISMA checklist

Section/topic	#	Checklist item	Reported on page #
**TITLE**			
Title	1	Identify the report as a systematic review, meta-analysis, or both.	1
**ABSTRACT**			
Structured summary	2	Provide a structured summary including, as applicable: background; objectives; data sources; study eligibility criteria, participants, and interventions; study appraisal and synthesis methods; results; limitations; conclusions and implications of key findings; systematic review registration number.	1
**INTRODUCTION**			
Rationale	3	Describe the rationale for the review in the context of what is already known.	2
Objectives	4	Provide an explicit statement of questions being addressed with reference to participants, interventions, comparisons, outcomes, and study design (PICOS).	2
**METHODS**			
Protocol and registration	5	Indicate if a review protocol exists, if and where it can be accessed (e.g., Web address), and, if available, provide registration information including registration number.	Not Applicable
Eligibility criteria	6	Specify study characteristics (e.g., PICOS, length of follow-up) and report characteristics (e.g., years considered, language, publication status) used as criteria for eligibility, giving rationale.	2
Information sources	7	Describe all information sources (e.g., databases with dates of coverage, contact with study authors to identify additional studies) in the search and date last searched.	3
Search	8	Present full electronic search strategy for at least one database, including any limits used, such that it	2

		could be repeated.	
Study selection	9	State the process for selecting studies (i.e., screening, eligibility, included in systematic review, and, if applicable, included in the meta-analysis).	3
Data collection process	10	Describe method of data extraction from reports (e.g., piloted forms, independently, in duplicate) and any processes for obtaining and confirming data from investigators.	3
Data items	11	List and define all variables for which data were sought (e.g., PICOS, funding sources) and any assumptions and simplifications made.	2
Risk of bias in individual studies	12	Describe methods used for assessing risk of bias of individual studies (including specification of whether this was done at the study or outcome level), and how this information is to be used in any data synthesis.	4
Summary measures	13	State the principal summary measures (e.g., risk ratio, difference in means).	3-4
Synthesis of results	14	Describe the methods of handling data and combining results of studies, if done, including measures of consistency (e.g., I^2) for each meta-analysis.	3-4
Risk of bias across studies	15	Specify any assessment of risk of bias that may affect the cumulative evidence (e.g., publication bias, selective reporting within studies).	4
Additional analyses	16	Describe methods of additional analyses (e.g., sensitivity or subgroup analyses, meta-regression), if done, indicating which were pre-specified.	4
**RESULTS**			
Study selection	17	Give numbers of studies screened, assessed for eligibility, and included in the review, with reasons for exclusions at each stage, ideally with a flow diagram.	4
Study characteristics	18	For each study, present characteristics for which data were extracted (e.g., study size, PICOS, follow-up period) and provide the citations.	4
Risk of bias within	19	Present data on risk of bias of each study and, if available, any outcome level assessment (see item 12).	5

studies			
Results of individual studies	20	For all outcomes considered (benefits or harms), present, for each study: (a) simple summary data for each intervention group (b) effect estimates and confidence intervals, ideally with a forest plot.	4-5
Synthesis of results	21	Present results of each meta-analysis done, including confidence intervals and measures of consistency.	4-5
Risk of bias across studies	22	Present results of any assessment of risk of bias across studies (see Item 15).	5-6
Additional analysis	23	Give results of additional analyses, if done (e.g., sensitivity or subgroup analyses, meta-regression [see Item 16]).	5
**DISCUSSION**			
Summary of evidence	24	Summarize the main findings including the strength of evidence for each main outcome; consider their relevance to key groups (e.g., healthcare providers, users, and policy makers).	6
Limitations	25	Discuss limitations at study and outcome level (e.g., risk of bias), and at review-level (e.g., incomplete retrieval of identified research, reporting bias).	8
Conclusions	26	Provide a general interpretation of the results in the context of other evidence, and implications for future research.	8
**FUNDING**			
Funding	27	Describe sources of funding for the systematic review and other support (e.g., supply of data); role of funders for the systematic review.	9

Supplementary Table 2. Characteristics of the studies included for the meta-analysis.

Author	Country	Sample size(MSC/control)	Species, strain	Gender	Methods of MS induction	MSC source	Dose	delivery route	Time of administration(dpi)	follow-up(days)
Abramowski, 2016 (1)	Germany	11/8	mice, C57BL/6	female	MOG_{35-55}, SC	BM-MSC, mice, syngeneic	1×10^6	IV	11	14
Bai, 2009 (2)	USA	6/12	mice, C57BL/6	female	MOG_{35-55}, SC	BM-MSC，human, xenogeneic	3×10^6	IV	16	45
		7/15							26	45
		10/12	mice, SJL	female	$PLP_{139-151}$, SC				16	60
Barati, 2019 (3)	Iran	10/10	mice, C57BL/6	male	cuprizone, oral	BM-MSC, mice, syngeneic	3×10^5	ICV	84	98
Barhum, 2010 (4)	Israel	16/25	mice, C3H.SW	female	MOG_{35-55}, SC	BM-MSC，human, xenogeneic	5×10^5	ICV	6	30
Boroujeni, 2020 (5)	Iran	10/10	mice, C57BL/6	male	cuprizone, oral	BM-MSC, mice, syngeneic	1×10^6	IN	84	114
Bravo, 2016 (6)	Spain	16/16	mice, C57BL/6	female	MOG_{35-55}, SC	DMSC, human, xenogeneic	1×10^6	IP	10, 14, 18, 20, 24	25
Constantin, 2009 (7)	Italy	10/10	mice, C57BL/6	female	MOG_{35-55}, SC	ASC, mice, syngeneic	1×10^6	IV	23, 28	72
Cruz-Martinez, 2016 (8)	Spain	6/6	mice, C57BL/6	NR	cuprizone, oral	BM-MSC, mice, syngeneic	3×10^6	ICV	84	90
Dang, 2014 (9)	China	7/8	mice, C57BL/6	female	MOG_{35-55}, SC	BM-MSC, mice, syngeneic	5×10^5	IV	10,15	30
Donders, 2014 (10)	Belgium	15/15	Rat, DA	female	MOG, SC	WJ-MSC, human, xenogeneic	2×10^6	IV	10-16	46
		11/9							28	46
Fathollahi, 2021 (11)	Iran	5/5	mice, C57BL/6	female	MOG_{35-55}, SC	ASC, mice, syngeneic	5×10^5	IN	15,24	27

Fisher-Shoval, 2012 (12)	Israel	10/11	mice, C57BL/6	female	MOG, SC	PMSC, human, xenogeneic	2×10^5	ICV	14	26
Fransson, 2014 (13)	Sweden	10/10	mice, C57BL/6	female	MOG_{35-55} , SC	BM-MSC, human, xenogeneic	1×10^4	IN	15	30
		10/10						IP		
Ganji, 2020 (14)	Iran	6/6	rat, Wistar	male	cuprizone, oral	ASC, human, xenogeneic	1×10^6	IV	21	49
Gerdoni, 2007 (15)	Italy	20/20	mice, SJL/J	female	PLP $_{139-151}$, SC	BM-MSC, mice, allogeneic	1×10^6	IV	12	72
Glenn, 2014 (16)	USA	3/3	mice, C57BL/6	female	MOG_{37-50} , SC	BM-MSC, mice, syngeneic	1×10^7	IP	3, 8	30
Gordon, 2008 (17)	UK	5/5	mice, C57BL/6	NR	MOG_{35-55} , SC	BM-MSC, human, xenogeneic	7.5×10^5	IP	6	50
Gordon, 2010 (18)	UK	10/10	mice, C57BL/6	NR	MOG_{35-55} , SC	BM-MSC, human, xenogeneic	1×10^6	IV	6	50
Gramlich, 2020 (19)	USA	20/20	mice, C57BL/6	female	MOG_{35-55} , SC	BM-MSC, human, xenogeneic	1×10^6	IV	7	32
Grigoriadis, 2011 (20)	Austria	11/8	mice, C57BL/6	female	MOG_{35-55} , SC (severe)	BM-MSC, mice, syngeneic	5×10^5	ICV	8-10	100
		9/8			MOG_{35-55} , SC (mild)					
Guo, 2013 (21)	China	15/15	mice, C57BL/6	female	MOG_{35-55} , SC	BM-MSC, human, xenogeneic	1×10^6	IV	12	26
Hao, 2016 (22)	China	11/11	rat, Wistar	female	GPSCH, SC	BM-MSC, rat, syngeneic	1×10^6	IV	10	32
Hedayatpour, 2012 (23)	Iran	3/3	mice, C57BL/6	male	cuprizone, oral	ASC, mice, syngeneic	1×10^6	IV	42	52
Hou, 2013 (24)	Korea	10/10	mice, C57BL/6	female	MOG_{35-55} , SC	BM-MSC, human, xenogeneic	1.5×10^6	IV	7	46

Hu, 2021 (25)	China	6/6	mice, C57BL/6	female	$MOG_{35\text{-}55}$, SC	BM-MSC, mice, syngeneic	4×10^6	IV	6, 8	25
Jafarinia, 2020 (26)	Iran	5/5	mice, C57BL/6	female	$MOG_{35\text{-}55}$, SC	ASC, human, xenogeneic	1×10^6	IV	10	30
Jaramillo-Mercha´n, 2013 (27)	spain	7/7	mice, C3H/He	NR	cuprizone, oral	BM-MSC, mice, allogeneic	3×10^5	ICP	90	105
Jiang, 2017 (28)	China	5/5	rat, Lewis	male	GPSCH, SC	PMSC, rat, allogeneic	1×10^6	ICV	10	56
		5/5				ES-MSC, rat, allogeneic				
Kashani, 2012 (29)	Iran	4/4	mice, C57BL/6	male	cuprizone, oral	ASC, mice, syngeneic	1×10^6	IV	42	52
Kassis, 2008 (30)	Israel	10/8	mice, C57BL/6	female	$MOG_{35\text{-}55}$, SC	BM-MSC, mice, syngeneic	1×10^6	IV	10	50
		10/12						ICV		
Kassis, 2013 (31)	Israel	10/5	mice, C57BL/6	female	$MOG_{35\text{-}55}$, SC	BM-MSC, mice (EAE), syngeneic	1×10^6	IV	10	40
		10/5				BM-MSC, mice (naïve), syngeneic				
Kassis, 2021 (32)	Israel	12/12	mice, C57BL/6	female	$MOG_{35\text{-}55}$, SC	BM-MSC, human, xenogeneic	5×10^5	ICV	8	40
Khezri, 2018 (33)	Iran	7/7	rat, Wistar	male	GPSCH, SC	BM-MSC, rat, syngeneic	2×10^6	IP	NR(symptoms onset)	37
Kim, 2017 (34)	Korea	9/9	mice, C57BL/6	female	$MOG_{35\text{-}55}$, SC	BM-MSC, human, xenogeneic	1×10^6	IV	14	46
Kim, 2018 (35)	Korea	3/3	mice, C57BL/6	female	$MOG_{35\text{-}55}$, SC	BM-MSC, human, xenogeneic	1×10^6	IV	14	50
Kurte, 2015 (36)	Chile	7/2	mice, C57BL/6	female	$MOG_{35\text{-}55}$, SC	BM-MSC, mice, syngeneic	1×10^6	IV	10	50
		7/2							18	50

		7/2							30	50
Kurte, 2020 (37)	Chile	12/12	mice, C57BL/6	female	MOG$_{35-55}$, SC	BM-MSC, mice, syngeneic	1×10^6	IP	7	30
Li-1, 2019 (38)	China	6/6	mice, C57BL/6	female	MOG$_{35-55}$, SC	UC-MSCs, human, xenogeneic	2×10^6	IV	12, 22	30
Li-2, 2019 (39)	China	8/8	rat, Sprague-Dawley	female	GPSCH, SC	BM-MSC, rat, syngeneic	1×10^6	IV	1	14
Liao, 2016 (40)	USA	6/6	mice, C57BL/6	NR	MOG$_{35-55}$, SC	BM-MSC, human, xenogeneic	1×10^6	IV	14	35
Liu, 2013 (41)	China	4/4	mice, C57BL/6	female	MOG$_{35-55}$, SC	UC-MSCs, human, xenogeneic	2×10^7	IV	15	50
Liu, 2020 (42)	China	9/9	mice, C57BL/6	female	MOG$_{35-55}$, SC	BM-MSC, mice, syngeneic	5×10^5	IV	11	18
Liu, 2021 (43)	China	15/15	mice, C57BL/6	male	MOG$_{35-55}$, SC	BM-MSC, mice, syngeneic	NR	ICV	8	36
Lu, 2009 (44)	China	9/14	mice, C57BL/6	female	MOG$_{35-55}$, SC	BM-MSC, human, xenogeneic	1×10^6	IV	10	30
Mahfouz, 2017 (45)	Egypt	15/15	mice, Swiss	male	GPSCH, SC	BM-MSC, mice, allogeneic	1×10^6	IP	0	28
Manganeli Polonio, 2021 (46)	Brazil	7/7	mice, C57BL/6	female	MOG$_{35-55}$, SC	meMSC, mice, syngeneic	2×10^6	IP	0, 10	30
Marin-Bañasco, 2014 (47)	Spain	9/9	mice, C57BL/6	female	MOG$_{35-55}$, SC	ASC, mice, syngeneic	1×10^6	IV	13	35
		10/9	mice, SJL/JCrl	female	PLP$_{139-151}$, SC	ASC, mice, syngeneic	1×10^6	IV	12	50
Marin-Bañasco, 2017 (48)	Spain	11/11	mice, SJL	female	PLP$_{139-151}$, SC	ASC, mice, syngeneic	1×10^6	IV	11	50
		8/9	mice, C57BL/6	female	MOG$_{35-55}$, SC	ASC, mice, allogeneic	1×10^6	IV	12	35

Marzban, 2018 (49)	Iran	12/12	mice, C57BL/6	male	cuprizone, oral	BM-MSC, human, xenogeneic	2×10^6	IP	28	42
Mitra, 2015 (50)	Malaysia	8/8	mice, C57BL/6	female	MOG_{35-55} , SC	BM-MSC, mice, syngeneic	5×10^5	IV	11	20
Morando, 2012 (51)	Italy	14/14	mice, C57BL/6	NR	MOG_{35-55} , SC	BM-MSC, human, xenogeneic	1×10^6	IT	10	45
		14/14						IV		
Payne-1, 2012 (52)	Australia	5/5	mice, C57BL/6	female	MOG_{35-55} , SC	BM-MSC, human, xenogeneic	1×10^6	IV	12, 14, 16	22
		5/5				ASC, human, xenogeneic				
		5/5				UC-MSC, human, xenogeneic				
Payne-2, 2012 (53)	Australia	10/10	mice, C57BL/6	female	MOG_{35-55} , SC	ASC, human, xenogeneic	5×10^6	IP	12, 13, 14	33
Payne, 2013 (54)	Australia	10/10	mice, C57BL/6	female	MOG_{35-55} , SC	ASC, human, xenogeneic	3×10^6	IP	1, 3, 5	40
Rafei-1, 2009 (55)	Canada	10/10	mice, Balb/c	NR	MOG, SC	BM-MSC, mice, syngeneic	2×10^6	IP	16, 28	50
		10/10				BM-MSC, mice, allogeneic				
Rafei-2, 2009 (56)	Canada	10/10	mice, Balb/c	female	MOG, SC	BM-MSC, mice, allogeneic	2×10^6	IP	15, 27, 45	46
Ryu, 2013 (57)	Korea	7/7	mice, C57BL/6	female	MOG_{35-55} , SC	BM-MSC, human, xenogeneic	1×10^6	IV	7	60
Schafer, 2008 (58)	Germany	8/8	rat, DA	female	MOG, SC	BM-MSC, rat, syngeneic	4×10^6	IV	0	40
Scruggs, 2013 (59)	USA	23/11	mice, C57BL/6	female	MOG_{35-55} , SC	ASC(young), human, xenogeneic	1×10^6	IP	0	30

		14/11				ASC(old), human, xenogeneic				30
Selim, 2016 (60)	Egypt	5/3	rat, Wistar	female	$MOG_{35\text{-}55}$, SC	PMSC, human, xenogeneic	1×10^6	IV	9	30
		5/3							16	30
Semon, 2014 (61)	USA	10/6	mice, C57BL/6	female	$MOG_{35\text{-}55}$, SC	BM-MSC, human, xenogeneic	1×10^6	IP	0	30
		12/6				ASC, human, xenogeneic				
Shalaby, 2016 (62)	Egypt	7/4	rat, albino	female	$MOG_{35\text{-}55}$, SC	ASC, human, xenogeneic	1×10^6	IV	15	40
		7/4							25	40
Shapira, 2016 (63)	Israel	20/20	mice, C57BL/6	female	$MOG_{35\text{-}55}$, SC	PMSC, human, xenogeneic	2×10^6	IM	11, 15	20
Shiri, 2020 (64)	Iran	3/3	mice, C57BL/6	male	cuprizone, oral	UC-MSC, human, xenogeneic	3×10^5	ICV	84	96
Shu, 2018 (65)	China	8/8	mice, C57BL/6	female	$MOG_{35\text{-}55}$, SC	AMC, human, xenogeneic	1×10^6	IP	14	35
Singh, 2017 (66)	India	6/6	mice, C57BL/6	male	$MOG_{35\text{-}55}$, SC	BM-MSC, mice, syngeneic	1×10^6	IV	8	38
Strong, 2015 (67)	USA	8/4	mice, C57BL/6	female	MOG, SC	lnASC, human, xenogeneic	1×10^6	IP	15	30
		7/4				obASC, human, xenogeneic				
Tahmasebi, 2020 (68)	Iran	5/5	mice, C57BL/6	male	cuprizone, oral	BM-MSC, mice, syngeneic	3×10^5	ICV	91	105
Togha, 2017 (69)	Iran	7/7	mice, C57BL/6	female	$MOG_{35\text{-}55}$, SC	BM-MSC, mice, syngeneic	2×10^6	IP	10, 17	50
Trubiani, 2016 (70)	Italy	10/10	mice, C57BL/6	male	MOG, SC	PDLCS, human, xenogeneic	1×10^6	IV	14	56

Vega-Letter, 2016 (71)	Chile	10/10	mice, C57BL/6	female	MOG_{35-55}, SC	BM-MSC, mice, syngeneic	2×10^6	IP	4	27
Wang, 2008 (72)	China	7/7	rat, Lewis	female	MBP_{68-84}, SC	BM-MSC, rat, syngeneic	1×10^7	IV	6	28
Wang, 2014 (73)	USA	6/6	mice, C57BL/6	NR	MOG_{35-55}, SC	ES-MSC, human, xenogeneic	1×10^6	IP	18	35
Wang, 2016 (74)	China	10/10	mice, C57BL/6	female	MOG_{35-55}, SC	BM-MSC, mice, syngeneic	1.5×10^6	IV	7	50
Wang, 2018 (75)	China	12/12	mice, C57BL/6	female	MOG_{35-55}, SC	UC-MSC, human, xenogeneic	5.5×10^6	IV	7, 14, 21, 28	33
Xin, 2020 (76)	China	3/3	mice, C57BL/6	female	MOG_{35-55}, SC	BM-MSC, mice, syngeneic	1×10^7	IP	14,20	42
Yousefi, 2013 (77)	Iran	8/4	mice, C57BL/6	female	MOG_{35-55}, SC	ASC, mice, syngeneic	0.5×10^6	IP	12	60
		8/4					1×10^6	IV	12	60
Yousefi, 2016 (78)	Iran	8/8	mice, C57BL/6	female	MOG_{35-55}, SC	ASC, mice, syngeneic	1×10^6	IP	10, 17, 21, 28	60
Yu, 2016 (79)	China	15/15	mice, C57BL/6	female	MOG_{35-55}, SC	BM-MSC, mice, syngeneic	1.5×10^5	IN	12	28
Zappia, 2005 (80)	Italy	8/4	mice, C57BL/6	female	MOG_{35-55}, SC	BM-MSC, mice, syngeneic	1×10^6	IV	10	48
		8/4							15	48
		6/6							24	60
Zhang, 2005 (81)	USA	10/3	mice, SJL/J	female	$PLP_{139-151}$, SC	BM-MSC, human, xenogeneic	0.5×10^6	IV	NR(symptoms onset)	+90
		10/3					2×10^6	IV	NR(symptoms onset)	+90
		10/3					3×10^6	IV	NR(symptoms onset)	+90
Zhang, 2006 (82)	USA	4/4	mice, SJL/J	female	$PLP_{139-151}$, SC	BM-MSC, human, xenogeneic	2×10^6	IV	NR(symptoms onset)	+ 315

Zhang, 2009 (83)	USA	8/15	mice, SJL/J	female	PLP $_{139-151}$, SC	BM-MSC, mice, syngeneic	2×10^6	IV	NR(symptoms onset)	+90
Zhang, 2014 (84)	China	20/10	mice, C57BL/6	female	MOG_{35-55} , SC	ASC, mice, syngeneic (EAE)	1×10^6	IP	0	30
		20/10				ASC, mice, syngeneic (wt)				
Zhang-1, 2020 (85)	China	8/8	rat, Wistar	female	GPSCH, SC	BM-MSC, rat, syngeneic	1×10^6	ICV	NR(symptoms onset)	+28
Zhang-2, 2020 (86)	China	20/20	mice, C57BL/6	female	MOG_{35-55} , SC	UC-MSC, human, xenogeneic	1×10^6	IV	13	42
Zhou, 2020 (87)	China	10/10	mice, C57BL/6	female	MOG_{35-55} , SC	UC-MSC, human, xenogeneic	1×10^7	IV	14	28
Zhu, 2012 (88)	China	10/5	mice, C57BL/6	female	MOG_{35-55} , SC	BM-MSC, mice, syngeneic	1×10^6	IV	20, 22	60
		10/5				BM-MSC, mice, allogeneic				

Legends: DPI: days post immunization; NR, not recorded; MOG, myelin oligodendrocyte glycoprotein; PLP, proteolipid protein; GPSCH, Guinea Pig Spinal Cord Homogenate; MBP, myelin basic protein; SC, subcutaneous; BM-MSC, bone marrow-derived mesenchymal stem cell; DMSC, decidua-derived mesenchymal stem cell; ASC, adipose tissue-derived mesenchymal stem cell; UC-MSC, umbilical cord mesenchymal stem cell; PMSC, placenta-derived mesenchymal stem cell; ES-MSC, embryonic stem cell-derived mesenchymal stem cell; PDLSC, periodontal ligament-derived mesenchymal stem cell; AMC, amnion mesenchymal stem cell; meMSC, murine endometrial-derived mesenchymal stem cell; IV, intravenous; IP, intraperitoneal; IN, intranasal; ICV, intra-cerebroventricular; IM, intramuscular; ICP, intra-cerebroparenchymal; IT, intrathecal.

Supplementary Table 3. Outcomes of the studies included for the meta-analysis.

Study	Clinical score	TEM	MBP staining	LFB staining	Spielmeyer staining	Solochrome Cyanin staining	HE staining	MRI	OPC	Olig	Morris water maze	Baaket test	Footprint analysis	Track visualizations	Rotarod
Abramowski, 2016	√														
Bai, 2009	√			√					√	√					
Barati, 2019		√		√						√					
Barhum, 2010	√														
Boroujeni, 2020		√		√						√	√				
Bravo, 2016	√														
Constantin, 2009	√				√				√						
Cruz-Martinez, 2016		√						√	√	√					
Dang, 2014	√														
Donders, 2014	√														
Fathollahi, 2021	√			√											
Fisher-Shoval, 2012	√														
Fransson, 2014	√														
Ganji, 2020		√	√							√		√			
Gerdoni, 2007	√														
Glenn, 2014	√														
Gordon, 2008	√														
Gordon, 2010	√		√												
Gramlich, 2020	√														
Grigoriadis, 2011	√														
Guo, 2013	√														
Hao, 2016	√			√											
Hedayatpour, 2012		√													
Hou, 2013	√		√	√											
Hu, 2021	√		√												
Jafarinia, 2020	√		√	√						√					
Jaramillo-Mercha´n, 2013			√						√						

Jiang, 2017	√		√							
Kashani, 2012		√						√		
Kassis, 2008	√									
Kassis, 2013	√			√						
Kassis, 2021	√									
Khezri, 2018						√				
Kim, 2017	√									
Kim, 2018	√		√	√						
Kurte, 2015	√									
Kurte, 2020	√									
Li-1, 2019	√			√						
Li-2, 2019	√			√						
Liao, 2016	√			√						
Liu, 2013				√						
Liu, 2020	√			√						
Liu, 2021	√			√						
Lu, 2009	√				√		√			
Mahfouz, 2017	√									
Manganeli Polonio, 2021	√			√						
Marin-Bañasco, 2014	√		√							
Marin-Bañasco 2017	√		√							
Marzban, 2018				√						
Mitra, 2015	√								√	
Morando, 2012	√			√						
Payne-1, 2012	√									
Payne-2, 2012	√									
Payne, 2013	√									
Rafei-1, 2009	√									
Rafei-2, 2009	√									
Ryu, 2013	√			√						
Schafer, 2008	√									
Scruggs, 2013	√			√						√
Selim, 2016	√									
Semon, 2014	√			√						

Shalaby, 2016	√							
Shapira, 2016	√							
Shiri, 2020		√		√		√		
Shu, 2018	√			√				
Singh, 2017	√			√				
Strong, 2015	√			√			√	
Tahmasebi, 2020		√		√		√		√
Togha, 2017	√			√				
Trubiani, 2016	√							
Vega-Letter, 2016	√							
Wang, 2008	√							
Wang, 2014	√							
Wang, 2016	√							
Wang, 2018	√	√	√	√				
Xin, 2020	√			√				
Yousefi, 2013	√							
Yousefi, 2016	√							
Yu, 2016	√			√				
Zappia, 2005				√				
Zhang, 2005	√			√				
Zhang, 2006	√							
Zhang, 2009	√			√				
Zhang, 2014	√			√				
Zhang-1, 2020	√				√			
Zhang-2, 2020	√		√	√				
Zhou, 2020	√							
Zhu, 2012	√							

Legends: TEM, transmission electron microscope; MBP, myelin basic protein; LFB, Luxol Fast Blue; HE, hematoxylin-eosin; MRI, magnetic resonance imaging; OPC, oligodendrocyte precursor cell; Olig, oligodendrocyte.

Supplementary Table 4. Quality scores of the studies included for the meta-analysis.

Study	(1)	(2)	(3)	(4)	(5)	(6)	(7)	(8)	(9)	(10)	Total
Abramowski, 2016	√					√		√		√	4
Bai, 2009	√					√		√			3
Barati, 2019	√		√			√		√	√	√	6
Barhum, 2010	√					√		√			3
Boroujeni, 2020	√					√		√	√		4
Bravo, 2016	√					√		√		√	4
Constantin, 2009	√					√		√		√	4
Cruz-Martinez, 2016	√			√		√		√		√	5
Dang, 2014	√					√		√		√	4
Donders, 2014	√		√			√		√		√	5
Fathollahi, 2021	√	√	√	√	√	√		√		√	8
Fisher-Shoval, 2012	√					√		√	√		4
Fransson, 2014	√				√	√		√		√	5
Ganji, 2020	√		√			√		√	√	√	6
Gerdoni, 2007	√				√	√		√			4
Glenn, 2014	√					√				√	3
Gordon, 2008	√					√					2
Gordon, 2010	√		√	√		√					4
Gramlich, 2020	√					√		√			3
Grigoriadis, 2011	√					√					2
Guo, 2013	√					√		√			3
Hao, 2016	√	√	√		√	√		√		√	7
Hou, 2013	√		√			√		√		√	5
Hu, 2021	√					√		√		√	4
Hedayatpour, 2012	√		√			√				√	4
Jafarinia, 2020	√					√		√		√	4
Jaramillo-Mercha´n, 2013	√				√	√		√		√	5
Jiang, 2017	√		√		√	√		√		√	6
Kashani, 2012	√		√			√					3
Kassis, 2008	√				√	√	√	√			5
Kassis, 2013	√		√		√	√				√	5
Kassis, 2021	√					√				√	3
Khezri, 2018	√		√		√	√		√	√		6
Kim, 2017	√		√		√	√		√	√	√	7
Kim, 2018	√		√		√	√	√	√		√	7
Kurte, 2015	√					√		√		√	4
Kurte, 2020	√					√		√		√	4

Liao, 2016	√				√	√		√		√	5
Li-1, 2019	√		√			√		√		√	5
Li-2, 2019	√		√	√	√	√		√		√	7
Liu, 2013	√					√		√		√	4
Liu, 2020	√				√	√		√		√	5
Liu, 2021	√		√	√	√	√	√	√		√	8
Lu, 2009	√					√		√			3
Mahfouz, 2017	√		√	√		√		√		√	6
Manganeli Polonio, 2021	√					√		√		√	4
Marin-Bañasco, 2014	√		√		√	√		√	√	√	7
Marin-Bañasco 2017	√		√		√	√		√	√	√	7
Marzban, 2018	√				√	√		√		√	5
Mitra, 2015	√					√		√		√	4
Morando, 2012	√					√				√	3
Payne-1, 2012	√					√		√			3
Payne-2, 2012	√					√		√		√	4
Payne, 2013	√					√		√			3
Rafei-1, 2009	√					√				√	3
Rafei-2, 2009	√					√				√	3
Ryu, 2013	√		√		√	√				√	5
Schafer, 2008	√					√		√			3
Scruggs, 2013	√		√	√	√	√		√		√	7
Selim, 2016	√				√	√		√		√	5
Semon, 2014	√				√	√		√		√	5
Shalaby, 2016	√					√		√		√	4
Shapira, 2016	√					√		√		√	4
Shiri, 2020	√		√		√	√		√		√	6
Shu, 2018	√		√		√	√		√	√	√	7
Singh, 2017	√			√	√	√		√		√	6
Strong, 2015	√					√		√		√	4
Tahmasebi, 2020	√				√	√		√	√	√	6
Togha, 2017	√				√	√		√	√	√	6
Trubiani, 2016	√		√			√	√	√	√	√	7
Vega-Letter, 2016	√					√		√		√	4
Wang, 2008	√					√		√			3
Wang, 2014	√		√	√	√	√		√			6
Wang, 2016	√		√			√	√	√		√	6
Wang, 2018	√		√			√		√		√	5
Xin, 2020	√		√			√		√		√	5
Yousefi, 2013	√				√	√		√			4
Yousefi, 2016	√				√	√		√			4
Yu, 2016	√				√	√	√	√	√	√	7

Zappia, 2005	√					√		√			3
Zhang, 2005	√		√		√	√		√			5
Zhang, 2006	√		√		√	√		√			5
Zhang, 2009	√		√		√	√		√			5
Zhang, 2016	√		√	√	√	√		√		√	7
Zhang-1, 2020	√		√		√	√				√	5
Zhang-2, 2020	√		√			√		√		√	5
Zhou, 2020	√					√		√	√	√	5
Zhu, 2012	√				√	√		√			4

Legends: (1) publication in a peer-reviewed journal; (2) reporting of a sample size calculation; (3) randomized treatment allocation; (4) allocation concealment; (5) blind assessment of outcome; (6) use of suitable animal models; (7) avoidance of anesthetics with significant intrinsic neuroprotective activity, such as ketamine; (8) statement of compliance with regulatory requirements; (9) statements describing temperature control; (10) declarations of potential conflicts of interest.

References

1. Abramowski P, Krasemann S, Ernst T, Lange C, Ittrich H, Schweizer M, et al. Mesenchymal Stromal/Stem Cells Do Not Ameliorate Experimental Autoimmune Encephalomyelitis and Are Not Detectable in the Central Nervous System of Transplanted Mice. *Stem Cells and Development* (2016) 25(15):1134-48. doi: 10.1089/scd.2016.0020.

2. Bai LH, Lennon DP, Eaton V, Maier K, Caplan AI, Miller SD, et al. Human Bone Marrow-Derived Mesenchymal Stem Cells Induce Th2-Polarized Immune Response and Promote Endogenous Repair in Animal Models of Multiple Sclerosis. *Glia* (2009) 57(11):1192-203. doi: 10.1002/glia.20841.

3. Barati S, Kashani IR, Tahmasebi F, Mehrabi S, Joghataei MT. Effect of mesenchymal stem cells on glial cells population in cuprizone induced demyelination model. *Neuropeptides* (2019) 75:75-84. doi: 10.1016/j.npep.2019.04.001.

4. Barhum Y, Gai-Castro S, Bahat-Stromza M, Barzilay R, Melamed E, Offen D. Intracerebroventricular Transplantation of Human Mesenchymal Stem Cells Induced to Secrete Neurotrophic Factors Attenuates Clinical Symptoms in a Mouse Model of Multiple Sclerosis. *J Mol Neurosci* (2010) 41(1):129-37. doi: 10.1007/s12031-009-9302-8.

5. Boroujeni FB, Pasbakhsh P, Mortezaee K, Pirhajati V, Alizadeh R, Aryanpour R, et al. Intranasal delivery of SDF-1 alpha-preconditioned bone marrow mesenchymal cells improves remyelination in the cuprizone-induced mouse model of multiple sclerosis. *Cell Biology International* (2020) 44(2):499-511. doi: 10.1002/cbin.11250.

6. Bravo B, Gallego MI, Flores AI, Bornstein R, Puente-Bedia A, Hernandez J, et al. Restrained Th17 response and myeloid cell infiltration into the central nervous system by human decidua-derived mesenchymal stem cells during experimental autoimmune encephalomyelitis. *Stem Cell Res Ther* (2016) 7. doi: 10.1186/s13287-016-0304-5.

7. Constantin G, Marconi S, Rossi B, Angiari S, Calderan L, Anghileri E, et al. Adipose-Derived Mesenchymal Stem Cells Ameliorate Chronic Experimental Autoimmune Encephalomyelitis. *Stem Cells* (2009) 27(10):2624-35. doi: 10.1002/stem.194.

8. Cruz-Martinez P, Gonzalez-Granero S, Molina-Navarro MM, Pacheco-Torres J, Garcia-Verdugo JM, Geijo-Barrientos E, et al. Intraventricular injections of mesenchymal stem cells activate endogenous functional remyelination in a chronic demyelinating murine model. *Cell Death Dis* (2016) 7. doi: 10.1038/cddis.2016.130.

9. Dang S, Xu H, Xu C, Cai W, Li Q, Cheng Y, et al. Autophagy regulates the therapeutic potential of mesenchymal stem cells in experimental autoimmune encephalomyelitis. *Autophagy* (2014) 10(7):1301-15. doi: 10.4161/auto.28771.

10. Donders R, Vanheusden M, Bogie JF, Ravanidis S, Thewissen K, Stinissen P, et al. Human Wharton's Jelly-Derived Stem Cells Display Immunomodulatory Properties and Transiently Improve Rat Experimental Autoimmune Encephalomyelitis. *Cell transplantation* (2015) 24(10):2077-98. Epub 2014/10/14. doi: 10.3727/096368914x685104.

11. Fathollahi A, Hashemi SM, Haji Molla Hoseini M, Tavakoli S, Farahani E, Yeganeh F. Intranasal administration of small extracellular vesicles derived from mesenchymal stem cells ameliorated the experimental autoimmune encephalomyelitis. *International Immunopharmacology* (2021) 90. doi: 10.1016/j.intimp.2020.107207.

12. Fisher-Shoval Y, Barhum Y, Sadan O, Yust-Katz S, Ben-Zur T, Lev N, et al. Transplantation of Placenta-Derived Mesenchymal Stem Cells in the EAE Mouse Model of MS. *J Mol Neurosci* (2012) 48(1):176-84. doi: 10.1007/s12031-012-9805-6.

13. Fransson M, Piras E, Wang H, Burman J, Duprez I, Harris RA, et al. Intranasal delivery of central nervous system-retargeted human mesenchymal stromal cells prolongs treatment efficacy of experimental autoimmune encephalomyelitis. *Immunology* (2014) 142(3):431-41. Epub 2014/03/05. doi: 10.1111/imm.12275.

14. Ganji R, Razavi S, Ghasemi N, Mardani M. Improvement of Remyelination in Demyelinated Corpus Callosum Using Human Adipose-Derived Stem Cells (hADSCs) and Pregnenolone in the Cuprizone Rat Model of Multiple Sclerosis. *Journal of molecular neuroscience : MN* (2020) 70(7):1088-99. Epub 2020/04/22. doi: 10.1007/s12031-020-01515-w.

15. Gerdoni E, Gallo B, Casazza S, Musio S, Bonanni I, Pedemonte E, et al. Mesenchymal stem cells effectively modulate pathogenic immune response in experimental autoimmune encephalomyelitis. *Ann Neurol* (2007) 61(3):219-27. doi: 10.1002/ana.21076.

16. Glenn JD, Smith MD, Calabresi PA, Whartenby KA. Mesenchymal Stem Cells Differentially Modulate Effector CD8+ T Cell Subsets and Exacerbate Experimental Autoimmune Encephalomyelitis. *Stem Cells* (2014) 32(10):2744-55. doi: 10.1002/stem.1755.

17. Gordon D, Pavlovska G, Glover CP, Uney JB, Wraith D, Scolding NJ. Human mesenchymal stem cells abrogate experimental allergic encephalomyelitis after intraperitoneal injection, and with sparse CNS infiltration. *Neurosci Lett* (2008) 448(1):71-3. doi: 10.1016/j.neulet.2008.10.040.

18. Gordon D, Pavlovska G, Uney JB, Wraith DC, Scolding NJ. Human Mesenchymal Stem Cells Infiltrate the Spinal Cord, Reduce Demyelination, and Localize to White Matter Lesions in Experimental Autoimmune Encephalomyelitis. *J Neuropath Exp Neur* (2010) 69(11):1087-95. doi: 10.1097/NEN.0b013e3181f97392.

19. Gramlich OW, Brown AJ, Godwin CR, Chimenti MS, Boland LK, Ankrum JA, et al. Systemic Mesenchymal Stem Cell Treatment Mitigates Structural and Functional Retinal Ganglion Cell Degeneration in a Mouse Model of Multiple Sclerosis. *Transl Vis Sci Techn* (2020) 9(8). doi: 10.1167/tvst.9.8.16.

20. Grigoriadis N, Lourbopoulos A, Lagoudaki R, Frischer JM, Polyzoidou E, Touloumi O, et al. Variable behavior and complications of autologous bone marrow mesenchymal stem cells transplanted in experimental autoimmune encephalomyelitis. *Experimental Neurology* (2011) 230(1):78-89. doi: 10.1016/j.expneurol.2011.02.021.

21. Guo Y, Chan KH, Lai WH, Siu CW, Kwan SC, Tse HF, et al. Human mesenchymal stem cells upregulate cd1dcd5+ regulatory b cells in experimental autoimmune encephalomyelitis. *NeuroImmunoModulation* (2013) 20(5):294-303. doi: 10.1159000351450105047.

22. Hao F, Li AP, Yu H, Liu MC, Wang YC, Liu J, et al. Enhanced Neuroprotective Effects of Combination Therapy with Bone Marrow-Derived Mesenchymal Stem Cells and Ginkgo biloba Extract (EGb761) in a Rat Model of Experimental Autoimmune Encephalomyelitis. *Neuroimmunomodulation* (2016) 23(1):41-57. doi: 10.1159/000437429.

23. Hedayatpour A, Ragerdi I, Pasbakhsh P, Kafami L, Atlasi N, Mahabadi VP, et al. Promotion of Remyelination by Adipose Mesenchymal Stem Cell Transplantation in A Cuptizone Model of Multiple Sclerosis. *Cell Journal* (2013) 15(2):142-51.

24. Hou Y, Ryu CH, Park KY, Kim SM, Jeong CH, Jeun SS. Effective combination of human bone marrow mesenchymal stem cells and minocycline in experimental autoimmune encephalomyelitis mice. *Stem Cell Res Ther* (2013) 4. doi: 10.1186/scrt228.

25. Hu RX, Lv WQ, Zhang SF, Liu YM, Sun B, Meng YT, et al. Combining miR-23b exposure with mesenchymal stem cell transplantation enhances therapeutic effects on EAE. *Immunology Letters* (2021) 229:18-26. doi: 10.1016/j.imlet.2020.11.007.

26. Jafarinia M, Alsahebfosoul F, Salehi H, Eskandari N, Azimzadeh M, Mahmoodi M, et al. Therapeutic effects of extracellular vesicles from human adipose-derived mesenchymal stem cells on chronic experimental autoimmune encephalomyelitis. *Journal of Cellular Physiology* (2020) 235(11):8779-90. doi: 10.1002/jcp.29721.

27. Jaramillo-Merchán J, Jones J, Ivorra JL, Pastor D, Viso-León MC, Armengól JA, et al. Mesenchymal stromal-cell transplants induce oligodendrocyte progenitor migration and remyelination in a chronic demyelination model. *Cell Death Dis* (2013) 4.

28. Jiang H, Zhang YY, Tian KW, Wang BB, Han S. Amelioration of experimental autoimmune encephalomyelitis through transplantation of placental derived mesenchymal stem cells. *Sci Rep-Uk* (2017) 7. doi: 10.1038/srep41837.

29. Kashani IR, Hedayatpour A, Pasbakhsh P, Kafami L, Atlasi N, Mahabadi VP, et al. 17β-estradiol enhances the efficacy of adipose-derived mesenchymal stem cells on remyelination in mouse model of multiple sclerosis. *Acta Medica Iranica* (2012) 50(12):789-97.

30. Kassis I, Grigoriadis N, Gowda-Kurkalli B, Mizrachi-Kol R, Ben-Hur T, Slavin S, et al. Neuroprotection and immunomodulation with mesenchymal stem cells in chronic experimental autoimmune encephalomyelitis. *Arch Neurol-Chicago* (2008) 65(6):753-61. doi: DOI 10.1001/archneur.65.6.753.

31. Kassis I, Petrou P, Halimi M, Karussis D. Mesenchymal stem cells (MSC) derived from mice with experimental autoimmune encephalomyelitis (EAE) suppress EAE and have similar biological properties with MSC from healthy donors. *Immunology Letters* (2013) 154(1-2):70-6. doi: 10.1016/j.imlet.2013.06.002.

32. Kassis I, Ben-Zwi M, Petrou P, Halimi M, Karussis D. Synergistic neuroprotective effects of Fingolimod and mesenchymal stem cells (MSC) in experimental autoimmune encephalomyelitis. *Immunology Letters* (2021). doi: 10.1016/j.imlet.2021.03.003.

33. Khezri S, Abtahi Froushani SM, Shahmoradi M. Nicotine Augments the Beneficial Effects of Mesenchymal Stem Cell-based Therapy in Rat Model of Multiple Sclerosis. *Immunological Investigations* (2018) 47(2):113-24. doi: 10.1080/08820139.2017.1391841.

34. Kim MJ, Lim JY, Park SA, Park SI, Kim WS, Ryu CH, et al. Effective combination of methylprednisolone and interferon beta-secreting mesenchymal stem cells in a model of multiple sclerosis. *Journal of Neuroimmunology* (2018) 314:81-8. doi: 10.1016/j.jneuroim.2017.11.010.

35. Kim MJ, Ryu CH, Kim SM, Lim JY, Kim WS, Jeun SS. Combined Treatment with Methylprednisolone and Human Bone Marrow-Derived Mesenchymal Stem Cells Ameliorate Experimental Autoimmune Encephalomyelitis. *Adv Exp Med Biol* (2018) 15(2):183-94. doi: 10.1007/s13770-017-0101-y.

36. Kurte M, Bravo-Alegria J, Torres A, Carrasco V, Ibanez C, Vega-Letter AM, et al. Intravenous Administration of Bone Marrow-Derived Mesenchymal Stem Cells Induces a Switch from Classical

to Atypical Symptoms in Experimental Autoimmune Encephalomyelitis. *Stem Cells International* (2015) 2015. doi: 10.1155/2015/140170.

37. Kurte M, Vega-Letter AM, Luz-Crawford P, Djouad F, Noel D, Khoury M, et al. Time-dependent LPS exposure commands MSC immunoplasticity through TLR4 activation leading to opposite therapeutic outcome in EAE. *Stem Cell Res Ther* (2020) 11(1). doi: 10.1186/s13287-020-01840-2.

38. Li H, Deng Y, Liang J, Huang F, Qiu W, Zhang M, et al. Mesenchymal stromal cells attenuate multiple sclerosis via IDO-dependent increasing the suppressive proportion of CD5+ IL-10+ B cells. *American Journal of Translational Research* (2019) 11(9):5673-88.

39. Li Z, Liu F, He X, Yang X, Shan F, Feng J. Exosomes derived from mesenchymal stem cells attenuate inflammation and demyelination of the central nervous system in EAE rats by regulating the polarization of microglia. *International Immunopharmacology* (2019) 67:268-80. doi: 10.1016/j.intimp.2018.12.001.

40. Liao W, Pham V, Liu L, Riazifar M, Pone EJ, Zhang SX, et al. Mesenchymal stem cells engineered to express selectin ligands and IL-10 exert enhanced therapeutic efficacy in murine experimental autoimmune encephalomyelitis. *Biomaterials* (2016) 77:87-97. doi: 10.1016/j.biomaterials.2015.11.005.

41. Liu R, Zhang Z, Lu Z, Borlongan C, Pan J, Chen J, et al. Human umbilical cord stem cells ameliorate experimental autoimmune encephalomyelitis by regulating immunoinflammation and remyelination. *Stem Cells and Development* (2013) 22(7):1053-62. doi: 10.1089/scd.2012.0463.

42. Liu Y, Ma Y, Du B, Wang Y, Yang GY, Bi X. Mesenchymal Stem Cells Attenuated Blood-Brain Barrier Disruption via Downregulation of Aquaporin-4 Expression in EAE Mice. *Molecular Neurobiology* (2020) 57(9):3891-901. doi: 10.1007/s12035-020-01998-z.

43. Liu GY, Wu Y, Kong FY, Ma S, Fu LY, Geng J. BMSCs differentiated into neurons, astrocytes and oligodendrocytes alleviated the inflammation and demyelination of EAE mice models. *PLoS One* (2021) 16(5):e0243014. Epub 2021/05/14. doi: 10.1371/journal.pone.0243014.

44. Lu ZQ, Hu XQ, Zhu CS, Wang DJ, Zheng XP, Liu QT. Overexpression of CNTF in Mesenchymal Stem Cells reduces demyelination and induces clinical recovery in experimental autoimmune encephalomyelitis mice. *Journal of Neuroimmunology* (2009) 206(1-2):58-69. doi: 10.1016/j.jneuroim.2008.10.014.

45. Mahfouz MM, Abdelsalam RM, Masoud MA, Mansour HA, Ahmed-Farid OA, Kenawy SA. The neuroprotective effect of mesenchymal stem cells on an experimentally induced model for multiple sclerosis in mice. *J Biochem Mol Toxic* (2017) 31(9). doi: 10.1002/jbt.21936.

46. C. MP, Longo de Freitas C, Garcia de Oliveira M, Rossato C, Nogueira Brandão W, Ghabdan Zanluqui N, et al. Murine endometrial-derived mesenchymal stem cells suppress experimental autoimmune encephalomyelitis depending on indoleamine-2,3-dioxygenase expression. *Clinical Science* (2021) 135(9):1065-82. doi: 10.1042/cs20201544.

47. Marin-Banasco C, Garcia MS, Guerrero IH, Sanchez RM, Estivill-Torrus G, Fernandez LL, et al. Mesenchymal properties of SJL mice-stem cells and their efficacy as autologous therapy in a relapsing-remitting multiple sclerosis model. *Stem Cell Res Ther* (2014) 5. doi: 10.1186/scrt524.

48. Marin-Banasco C, Benabdellah K, Melero-Jerez C, Oliver B, Pinto-Medel MJ, Hurtado-Guerrero I, et al. Gene therapy with mesenchymal stem cells expressing IFN-ss ameliorates

neuroinflammation in experimental models of multiple sclerosis. *Brit J Pharmacol* (2017) 174(3):238-53. doi: 10.1111/bph.13674.

49. Marzban M, Mousavizadeh K, Bakhshayesh M, Vousooghi N, Vakilzadeh G, Torkaman-Boutorabi A. Effect of multiple intraperitoneal injections of human bone marrow mesenchymal stem cells on cuprizone model of multiple sclerosis. *Iranian Biomedical Journal* (2015) 22(5):312-21. doi: 10.29252/ibj.22.5.312.

50. Mitra NK, Bindal U, Eng Hwa W, Chua CL, Tan CY. Evaluation of locomotor function and microscopic structure of the spinal cord in a mouse model of experimental autoimmune encephalomyelitis following treatment with syngeneic mesenchymal stem cells. *International journal of clinical and experimental pathology* (2015) 8(10):12041-52.

51. Morando S, Vigo T, Esposito M, Casazza S, Novi G, Principato M, et al. The therapeutic effect of mesenchymal stem cell transplantation in experimental autoimmune encephalomyelitis is mediated by peripheral and central mechanisms. *Stem Cell Research and Therapy* (2012) 3(1). doi: 10.1186/scrt94.

52. Payne NL, Sun G, McDonald C, Layton D, Moussa L, Emerson-Webber A, et al. Distinct immunomodulatory and migratory mechanisms underpin the therapeutic potential of human mesenchymal stem cells in autoimmune demyelination. *Cell transplantation* (2013) 22(8):1409-25. Epub 2012/10/13. doi: 10.3727/096368912x657620.

53. Payne NL, Dantanarayana A, Sun G, Moussa L, Caine S, McDonald C, et al. Early intervention with gene-modified mesenchymal stem cells overexpressing interleukin-4 enhances anti-inflammatory responses and functional recovery in experimental autoimmune demyelination. *Cell adhesion & migration* (2012) 6(3):179-89. Epub 2012/05/10. doi: 10.4161/cam.20341.

54. Payne NL, Sun G, McDonald C, Moussa L, Emerson-Webber A, Loisel-Meyer S, et al. Human adipose-derived mesenchymal stem cells engineered to secrete IL-10 inhibit APC function and limit CNS autoimmunity. *Brain, behavior, and immunity* (2013) 30:103-14. Epub 2013/02/02. doi: 10.1016/j.bbi.2013.01.079.

55. Rafei M, Birman E, Forner K, Galipeau J. Allogeneic Mesenchymal Stem Cells for Treatment of Experimental Autoimmune Encephalomyelitis. *Molecular Therapy* (2009) 17(10):1799-803. doi: 10.1038/mt.2009.157.

56. Rafei M, Campeau PA, Aguilar-Mahecha A, Buchanan M, Williams P, Birman E, et al. Mesenchymal Stromal Cells Ameliorate Experimental Autoimmune Encephalomyelitis by Inhibiting CD4 Th17 T Cells in a CC Chemokine Lisgand 2-Dependent Manner. *Journal of Immunology* (2009) 182(10):5994-6002. doi: 10.4049/jimmunol.0803962.

57. Ryu CH, Park KY, Hou Y, Jeong CH, Kim SM, Jeun SS. Gene Therapy of Multiple Sclerosis Using Interferon beta-Secreting Human Bone Marrow Mesenchymal Stem Cells. *Biomed Res Int* (2013) 2013. doi: 10.1155/2013/696738.

58. Schafer R, Ayturan M, Bantleon R, Kehlbach R, Siegel G, Pintaske J, et al. The Use of Clinically Approved Small Particles of Iron Oxide (SPIO) for Labeling of Mesenchymal Stem Cells Aggravates Clinical Symptoms in Experimental Autoimmune Encephalomyelitis and Influences Their In Vivo Distribution. *Cell Transplant* (2008) 17(8):923-41. doi: Doi 10.3727/096368908786576480.

59. Scruggs BA, Semon JA, Zhang X, Zhang S, Bowles AC, Pandey AC, et al. Age of the donor reduces the ability of human adipose-derived stem cells to alleviate symptoms in the experimental

autoimmune encephalomyelitis mouse model. *Stem cells translational medicine* (2013) 2(10):797-807. Epub 2013/09/11. doi: 10.5966/sctm.2013-0026.

60. Selim AO, Selim SA, Shalaby SM, Mosaad H, Saber T. Neuroprotective effects of placenta-derived mesenchymal stromal cells in a rat model of experimental autoimmune encephalomyelitis. *Cytotherapy* (2016) 18(9):1100-13. doi: 10.1016/j.jcyt.2016.06.002.

61. Semon JA, Maness C, Zhang X, Sharkey SA, Beuttler MM, Shah FS, et al. Comparison of human adult stem cells from adipose tissue and bone marrow in the treatment of experimental autoimmune encephalomyelitis. *Stem cell research & therapy* (2014) 5(1):2. Epub 2014/01/11. doi: 10.1186/scrt391.

62. Shalaby SM, Sabbah NA, Saber T, Abdel Hamid RA. Adipose-derived mesenchymal stem cells modulate the immune response in chronic experimental autoimmune encephalomyelitis model. *IUBMB Life* (2016) 68(2):106-15. doi: 10.1002/iub.1469.

63. Shapira I, Fainstein N, Tsirlin M, Stav I, Volinsky E, Moresi C, et al. Placental Stromal Cell Therapy for Experimental Autoimmune Encephalomyelitis: The Role of Route of Cell Delivery. *Stem Cell Transl Med* (2017) 6(4):1286-94. doi: 10.5966/sctm.2015-0363.

64. Shiri E, Pasbakhsh P, Borhani-Haghighi M, Alizadeh Z, Nekoonam S, Mojaverrostami S, et al. Mesenchymal Stem Cells Ameliorate Cuprizone-Induced Demyelination by Targeting Oxidative Stress and Mitochondrial Dysfunction. *Cell Mol Neurobiol* (2020). doi: 10.1007/s10571-020-00910-6.

65. Shu J, He X, Li H, Liu X, Qiu X, Zhou T, et al. The beneficial effect of human amnion mesenchymal cells in inhibition of inflammation and induction of neuronal repair in EAE mice. *Journal of Immunology Research* (2018) 2018. doi: 10.1155/2018/5083797.

66. Singh SP, Jadhav SH, Chaturvedi CP, Nityanand S. Therapeutic efficacy of multipotent adult progenitor cells versus mesenchymal stem cells in experimental autoimmune encephalomyelitis. *Regenerative Medicine* (2017) 12(4):377-96. doi: 10.2217/rme-2016-0109.

67. Strong AL, Bowles AC, Wise RM, Morand JP, Dutreil MF, Gimble JM, et al. Human Adipose Stromal/Stem Cells from Obese Donors Show Reduced Efficacy in Halting Disease Progression in the Experimental Autoimmune Encephalomyelitis Model of Multiple Sclerosis. *Stem Cells* (2016) 34(3):614-26. doi: 10.1002/stem.2272.

68. Tahmasebi F, Pasbakhsh P, Barati S, Madadi S, Kashani IR. The effect of microglial ablation and mesenchymal stem cell transplantation on a cuprizone-induced demyelination model. *J Cell Physiol* (2021) 236(5):3552-64. doi: 10.1002/jcp.30090.

69. Togha M, Jahanshahi M, Alizadeh L, Jahromi SR, Vakilzadeh G, Alipour B, et al. Rapamycin Augments Immunomodulatory Properties of Bone Marrow-Derived Mesenchymal Stem Cells in Experimental Autoimmune Encephalomyelitis. *Molecular Neurobiology* (2017) 54(4):2445-57. doi: 10.1007/s12035-016-9840-3.

70. Trubiani O, Giacoppo S, Ballerini P, Diomede F, Piattelli A, Bramanti P, et al. Alternative source of stem cells derived from human periodontal ligament: a new treatment for experimental autoimmune encephalomyelitis. *Stem Cell Res Ther* (2016) 7. doi: 10.1186/s13287-015-0253-4.

71. Vega-Letter AM, Kurte M, Fernandez-O'Ryan C, Gauthier-Abeliuk M, Fuenzalida P, Moya-Uribe I, et al. Differential TLR activation of murine mesenchymal stem cells generates distinct immunomodulatory effects in EAE. *Stem Cell Res Ther* (2016) 7. doi: 10.1186/s13287-016-0402-4.

72. Wang JH, Wang GY, Sun B, Li HL, Mu L, Wang Q, et al. Interleukin-27 suppresses experimental autoimmune encephalomyelitis during bone marrow stromal cell treatment. *J Autoimmun* (2008) 30(4):222-9. doi: 10.1016/j.jaut.2007.10.001.

73. Wang X, Kimbrel EA, Ijichi K, Paul D, Lazorchak AS, Chu J, et al. Human ESC-derived MSCs outperform bone marrow MSCs in the treatment of an EAE model of multiple sclerosis. *Stem Cell Reports* (2014) 3(1):115-30. doi: 10.1016/j.stemcr.2014.04.020.

74. Wang D, Li SP, Fu JS, Bai L, Guo L. Resveratrol augments therapeutic efficiency of mouse bone marrow mesenchymal stem cell-based therapy in experimental autoimmune encephalomyelitis. *Int J Dev Neurosci* (2016) 49:60-6. doi: 10.1016/j.ijdevneu.2016.01.005.

75. Wang YL, Xue P, Xu CY, Wang Z, Liu XS, Hua LL, et al. SPK1-transfected UCMSC has better therapeutic activity than UCMSC in the treatment of experimental autoimmune encephalomyelitis model of Multiple sclerosis. *Sci Rep-Uk* (2018) 8. doi: 10.1038/s41598-018-19703-5.

76. Xin Y, Gao J, Hu R, Li H, Li Q, Han F, et al. Changes of immune parameters of T lymphocytes and macrophages in EAE mice after BM-MSCs transplantation. *Immunology Letters* (2020) 225:66-73. doi: 10.1016/j.imlet.2020.05.005.

77. Yousefi F, Ebtekar M, Soleimani M, Soudi S, Hashemi SM. Comparison of in vivo immunomodulatory effects of intravenous and intraperitoneal administration of adipose-tissue mesenchymal stem cells in experimental autoimmune encephalomyelitis (EAE). *International Immunopharmacology* (2013) 17(3):608-16. doi: 10.1016/j.intimp.2013.07.016.

78. Yousefi F, Ebtekar M, Soudi S, Soleimani M, Hashemi SM. In vivo immunomodulatory effects of adipose-derived mesenchymal stem cells conditioned medium in experimental autoimmune encephalomyelitis. *Immunology Letters* (2016) 172:94-105. doi: 10.1016/j.imlet.2016.02.016.

79. Yu JW, Li YH, Song GB, Yu JZ, Liu CY, Liu JC, et al. Synergistic and Superimposed Effect of Bone Marrow-Derived Mesenchymal Stem Cells Combined with Fasudil in Experimental Autoimmune Encephalomyelitis. *J Mol Neurosci* (2016) 60(4):486-97. doi: 10.1007/s12031-016-0819-3.

80. Zappia E, Casazza S, Pedemonte E, Benvenuto F, Bonanni I, Gerdoni E, et al. Mesenchymal stem cells ameliorate experimental autoimmune encephalomyelitis inducing T-cell anergy. *Blood* (2005) 106(5):1755-61. doi: 10.1182/blood-2005-04-1496.

81. Zhang J, Li Y, Chen JL, Cui YS, Lu M, Elias SB, et al. Human bone marrow stromal cell treatment improves neurological functional recovery in EAE mice. *Experimental Neurology* (2005) 195(1):16-26. doi: 10.1016/j.expneurol.2005.03.018.

82. Zhang J, Li Y, Lu M, Cui YS, Chen JL, Noffsinger L, et al. Bone marrow stromal cells reduce axonal loss in experimental autoimmune encephalomyelitis mice. *Journal of Neuroscience Research* (2006) 84(3):587-95. doi: 10.1002/jnr.20962.

83. Zhang J, Brodie C, Li Y, Zheng XG, Roberts C, Lu M, et al. Bone marrow stromal cell therapy reduces proNGF and p75 expression in mice with experimental autoimmune encephalomyelitis. *Journal of the Neurological Sciences* (2009) 279(1-2):30-8. doi: 10.1016/j.jns.2008.12.033.

84. Zhang X, Bowles AC, Semon JA, Scruggs BA, Zhang S, Strong AL, et al. Transplantation of autologous adipose stem cells lacks therapeutic efficacy in the experimental autoimmune encephalomyelitis model. *PLoS One* (2014) 9(1):e85007. Epub 2014/01/28. doi: 10.1371/journal.pone.0085007.

85. Zhang JM, Wang H, Fan YY, Yang FH. Effect of mesenchymal stem cells transplantation on the changes of oligodendrocyte lineage in rat brain with experimental autoimmune encephalomyelitis. *Brain Behav* (2021) 11(2). doi: 10.1002/brb3.1999.

86. Zhang L, Wang X, Lu X, Ma Y, Xin X, Xu X, et al. Tetramethylpyrazine enhanced the therapeutic effects of human umbilical cord mesenchymal stem cells in experimental autoimmune encephalomyelitis mice through Nrf2/HO-1 signaling pathway. *Stem Cell Research and Therapy* (2020) 11(1). doi: 10.1186/s13287-020-01700-z.

87. Zhou X, Liu X, Liu L, Han C, Xie Z, Liu X, et al. Transplantation of IFN-γ Primed hUCMSCs Significantly Improved Outcomes of Experimental Autoimmune Encephalomyelitis in a Mouse Model. *Neurochemical Research* (2020) 45(7):1510-7. doi: 10.1007/s11064-020-03009-y.

88. Zhu J, Zhang J, Li Q, Du Y, Qiao B, Hu X. Transplanting of Mesenchymal Stem Cells May Affect Proliferation and Function of CD4+T Cells in Experimental Autoimmune Encephalomyelitis. *Experimental and Clinical Transplantation* (2012) 10(5):492-500. doi: 10.6002/ect.2011.0197.
